# Toe‐brachial index and toe systolic blood pressure for the diagnosis of peripheral arterial disease

**DOI:** 10.1002/14651858.CD013783.pub2

**Published:** 2024-10-30

**Authors:** Peta E Tehan, Joseph Mills, Sarah Leask, Christopher Oldmeadow, Benjamin Peterson, Mathew Sebastian, Viv Chuter

**Affiliations:** Department of Surgery, Sub-faculty of Clinical and Molecular Sciences, Faculty of Medicine, Nursing and Health SciencesMonash UniversityClaytonAustralia; Division of Vascular Surgery and Endovascular TherapyMichael E. DeBakey Department of Surgery, Baylor College of MedicineHoustonTexasUSA; Hunter Medical Research InsituteNew Lambton HeightsAustralia; The University of NewcastleCallaghanAustralia; Department of Podiatry, School of Health, Medical and Applied SciencesCQUniversityRockhamptonAustralia; Department of SurgeryJohn Hunter HospitalNew LambtonAustralia; School of Health SciencesWestern Sydney UniversityCampbelltownAustralia

## Abstract

**Background:**

Peripheral arterial disease (PAD) of the lower limbs is caused by atherosclerotic occlusive disease in which narrowing of arteries reduces blood flow to the lower limbs. PAD is common; it is estimated to affect 236 million individuals worldwide. Advanced age, smoking, hypertension, diabetes and concomitant cardiovascular disease are common factors associated with increased risk of PAD. Complications of PAD can include claudication pain, rest pain, wounds, gangrene, amputation and increased cardiovascular morbidity and mortality. It is therefore clinically important to use diagnostic tests that accurately identify PAD. Accurate and timely detection of PAD allows clinicians to implement appropriate risk management strategies to prevent complications, slow progression or intervene when indicated. Toe‐brachial index (TBI) and toe systolic blood pressure (TSBP) are amongst a suite of non‐invasive bedside tests used to detect PAD. Both TBI and TSBP are commonly utilised by a variety of clinicians in different settings, therefore a systematic review and meta‐analysis of their diagnostic accuracy is warranted and highly relevant to inform clinical practice.

**Objectives:**

To (1) estimate the accuracy of TSBP and TBI for the diagnosis of PAD in the lower extremities at different cut‐off values for test positivity in populations at risk of PAD, and (2) compare the accuracy of TBI and TSBP for the diagnosis of PAD in the lower extremities.

Secondary objectives were to investigate several possible sources of heterogeneity in test accuracy, including the following: patient group tested (people with type 1 or type 2 diabetes, people with renal disease and general population), type of equipment used, positivity threshold and type of reference standard.

**Search methods:**

The Cochrane Vascular Information Specialist searched the MEDLINE, Embase, CINAHL, Web of Science, LILACS, Zetoc and DARE databases and the World Health Organization International Clinical Trials Registry Platform and ClinicalTrials.gov trials registers to 27 February 2024.

**Selection criteria:**

We included diagnostic case‐control, cross‐sectional, prospective and retrospective studies in which all participants had either a TSBP or TBI measurement plus a validated method of vascular diagnostic imaging for PAD. We needed to be able to cross‐tabulate (2 x 2 table) results of the index test and the reference standard to include a study. To be included, study populations had to be adults aged 18 years and over. We included studies of symptomatic and asymptomatic participants. Studies had to use TSBP and TBI (also called toe‐brachial pressure index (TBPI)), either individually, or in addition to other non‐invasive tests as index tests to diagnose PAD in individuals with suspected disease. We included data collected by photoplethysmography, laser Doppler, continuous wave Doppler, sphygmomanometers (both manual and aneroid) and manual or automated digital equipment.

**Data collection and analysis:**

Two review authors independently completed data extraction using a standardised form. We extracted data to populate 2 x 2 contingency tables when available (true positives, true negatives, false positives, false negatives). Where data were not available to enable statistical analysis, we contacted study authors directly.

Two review authors working independently undertook quality assessment using QUADAS‐2, with disagreements resolved by a third review author. We incorporated two additional questions into the quality appraisal to aid our understanding of the conduct of studies and make appropriate judgements about risk of bias and applicability.

**Main results:**

Eighteen studies met the inclusion criteria; 13 evaluated TBI only, one evaluated TSBP only and four evaluated both TBI and TSBP. Thirteen of the studies used colour duplex ultrasound (CDU) as a reference standard, two used computed tomography angiography (CTA), one used multi‐detector row tomography (MDCT), one used angiography and one used a combination of CDU, CTA and angiography. TBI was investigated in 1927 participants and 2550 limbs. TSBP was investigated in 701 participants, of which 701 limbs had TSBP measured. Studies were generally of low methodological quality, with poor reporting of participant recruitment in regard to consecutive or random sampling, and poor reporting of blinding between index test and reference standard, as well as timing between index test and reference standard. The certainty of evidence according to GRADE for most studies was very low.

**Authors' conclusions:**

Whilst a small number of diagnostic test accuracy studies have been completed for TBI and TSBP to identify PAD, the overall methodological quality was low, with most studies providing a very low certainty of evidence. The evidence base to support the use of TBI and TSBP to identify PAD is therefore limited. Whilst both TBI and TSBP are used extensively clinically, the overall diagnostic performance of these tests remains uncertain. Future research using robust methods and clear reporting is warranted to comprehensively determine the diagnostic test accuracy of the TBI and TSBP for identification of PAD with greater certainty. However, conducting such research where some of the reference tests are invasive and only clinically indicated in populations with known PAD is challenging.

## Plain language summary

**How accurate are toe‐brachial index and toe systolic blood pressures for peripheral arterial disease?**

**Key messages**

‐ There is currently a lack of quality evidence to support the use of toe‐brachial index (TBI) and toe systolic blood pressure (TSBP) to detect peripheral arterial disease, and clinicians should interpret these tests with caution.

‐ More high‐quality studies with larger numbers of participants are required to increase the certainty of evidence.

**Why is improving the diagnosis of peripheral arterial disease important?**

Peripheral arterial disease affects approximately 236 million people globally. In peripheral arterial disease, the arteries that supply blood flow to the legs and feet are affected by fatty deposits (atherosclerosis), which leads to narrowing and can cause blockages. This can result in pain, wounds and gangrene, which can lead to amputation. Not recognising peripheral arterial disease when it is present (a false‐negative test result) may result in the condition not being appropriately treated or a delay in treatment. This could lead to increased rates of wounds, amputation and premature death. An incorrect diagnosis of peripheral arterial disease (a false‐positive result) may result in unnecessary imaging or inappropriate prescription of cardiovascular risk‐reducing medications where there is no benefit to be gained and the potential for side effects. An incorrect diagnosis may also result in stress and anxiety for the patient.

**What is the toe‐brachial index and toe systolic blood pressure?**

Clinicians have a variety of tests available to detect peripheral arterial disease, which are non‐invasive and can be performed by the bedside. Two of these tests are the toe‐brachial index (TBI) and toe systolic blood pressure (TSBP). TSBP is a blood pressure taken at the great or second toe. A small blood pressure cuff is wrapped around the toe and a Doppler, laser or photoplethysmograph probe is placed on the toe to detect a blood flow signal. The cuff is then inflated until the signal disappears, and then slowly deflated until the signal re‐appears. The pressure at which the signal returns is considered the TSBP. To measure the TBI, the TSBP is simply divided by the highest blood pressure of both arms, resulting in an index. Lower values indicate that peripheral arterial disease is present. A variety of different thresholds are used in practice and research.

**What did we want to find out?**

We wanted to find out how accurate the TBI and TSBP are for detecting peripheral arterial disease in people at risk of the condition. We also wanted to compare the accuracy of the TBI with TSBP.

**What did we do?**

We searched for studies that had investigated the accuracy of either TBI or TSBP for peripheral arterial disease, and examined the quality and certainty of these studies.

**What did we find?**

We found 18 studies: 13 evaluated TBI only, one evaluated TSBP only and four evaluated both TBI and TSBP. TBI was evaluated in 17 studies including 1927 participants and TSBP was evaluated in five studies including 701 participants. Studies were conducted in the USA, Australia, the UK, Japan, Korea, the Czech Republic, France, Hungary, India and Iran. The mean reported age range of participants was between 63 and 83 years. The studies were carried out in people who were at risk of peripheral arterial disease, such as people with diabetes, kidney disease, older people or people with known risk factors. However, there were not enough studies and the studies we found were too different from each other to allow a reliable estimate of the overall accuracy of the tests for diagnosing peripheral arterial disease.

**What are the limitations of the evidence?**

Only a small number of studies were included (18) and the studies were quite different from each other. This is because our inclusion criteria were broad and inclusive. However, this made it impossible to directly compare or pool (combine) the studies together. The studies that we included were generally of low quality, and we have low or very low confidence in the results. The included studies also had some important limitations in the way that they were conducted. This may have resulted in TSBP and the TBI appearing more accurate than they really are in some of the included studies.

**How up‐to‐date is this evidence?**

This review was conducted of all literature published up to 27 February 2024.

## Summary of findings

**Summary of findings 1:** Summary of findings table

**Accuracy of toe‐brachial index (TBI) in diagnosing peripheral arterial disease (PAD)**				
Population	People at risk of PAD, including the general population with risk factors, older people, renal disease or diabetes			
Setting	Primary or secondary care settings			
Index test	Toe‐brachial index			
Importance	In order to manage PAD effectively, a diagnosis should be accurately determined. It is therefore important that available, non‐invasive clinical tests are accurate to allow detection and subsequent management.			
Reference standard	Valid diagnostic imaging (duplex ultrasonography, digital subtraction angiography, computed tomography angiography or multi‐detector row computed tomography)			
Studies	16 observational prospective and 1 observational retrospective			
Quality (QUADAS‐2^a^) and comments	Most included studies had an unclear risk of bias. Some studies exhibited high risk of bias in relation to reporting of patient selection (random or consecutive sampling), reporting of blinding of the index test and reference standard, and reporting of timing between the index and reference standard in flow and timing. Applicability of patient selection was also an issue in some studies where the study population had either very low levels of PAD or very high levels of PAD. One study utilised a data‐driven approach to determine the diagnostic threshold that was not pre‐specified, which was a high risk for potential bias. Another study did not pre‐specify a threshold and reported on a number of different thresholds, which introduced bias.			
Study		Sensitivity (95% CI)	Specificity (95% CI)	GRADE certainty of evidence
AbuRahma 2020		0.85 (0.79 to 0.90)	0.62 (0.53 to 0.70)	VERY LOW
Babaei 2020		1.0 (0.75 to 1.0)	0.96 (0.94 to 0.97)	VERY LOW
Chen 2021		0.41 (0.24 to 0.59)	0.93 (0.88 to 0.97)	VERY LOW
Cleofort 2023		0.80 (0.69 to 0.89)	0.44 (0.27 to 0.62)	VERY LOW
Fejfarova 2021		0.97 (0.88 to 1.0)	0.47 (0.32 to 0.62)	VERY LOW
Fendrik 2023		0.91 (0.81 to 0.97) (MESI) 0.91 (0.81 to 0.97) (Systoe) 0.95 (0.86 to 0.99) (Periflux)	0.67 (0.60 to 0.74) (MESI) 0.77 (0.70 to 0.83) (Systoe) 0.76 (0.69 to 0.82) Periflux	LOW
Machaczka 2021		0.88 (0.69 to 0.97)	0.88 (0.64 to 0.99)	VERY LOW
Normahani 2020		0.60 (0.51 to 0.68)	0.86 (0.77 to 0.92)	LOW
Okamoto 2006		0.46 (0.31 to 0.61)	1.0 (0.87 to 1.0)	VERY LOW
Park 2012		1.0 (0.75 to 1.0)	1.0 (0.80 to 1.0)	VERY LOW
Singhania 2024		0.79 (0.63 to 0.90)	0.95 (0.90 to 0.98)	LOW
Sonter 2017		0.71 (0.54 to 0.85)	0.77 (0.63 to 0.87)	VERY LOW
Tehan 2016		0.70 (0.56 to 0.82)	0.79 (0.67 to 0.89)	LOW
Tehan 2021		0.81 (0.61 to 0.93)	0.75 (0.53 to 0.90)	VERY LOW
Tsuyuki 2013		0.81 (0.54 to 0.96)	0.63 (0.35 to 0.85)	VERY LOW
Vriens 2018		0.90 (0.68 to 0.99)	0.45 (0.29 to 0.62)	VERY LOW
Williams 2005		0.94 (0.81 to 0.99)	0.68 (0.57 to 0.77)	VERY LOW
**Accuracy of toe systolic blood pressure (TSBP) in diagnosing peripheral arterial disease (PAD)**				
Population	Populations at risk of PAD, including the general population with risk factors, and diabetes populations			
Setting	Primary or secondary care settings			
Index test	Toe systolic blood pressure			
Importance	In order to manage PAD effectively, a diagnosis should be accurately determined. It is therefore important that available, non‐invasive clinical tests are accurate to allow detection and subsequent management.			
Reference Standard	Colour duplex ultrasound			
Studies	4 observational prospective and 1 observational retrospective			
Quality (QUADAS‐2^a^) and comments	Most included studies had an unclear risk of bias. Two studies had a high risk of bias for the index test, with a lack of blinding reported; two other studies did not report blinding. One study had a high risk of bias for applicability of patient selection with very low PAD prevalence. One study utilised a data‐driven diagnostic threshold that was not pre‐specified, which will have resulted in a high potential for bias.			
Study		Sensitivity	Specificity	GRADE certainty of evidence
Fejfarova 2021		0.68 (0.57 to 0.80)	0.79 (0.64 to 0.89)	VERY LOW
Sonter 2017		0.44 (0.28 to 0.60)	0.98 (0.90 to 1.0)	VERY LOW
Tehan 2017		0.70 (0.64 to 0.75)	0.71 (0.62 to 0.79)	LOW
Tehan 2021		0.15 (0.04 to 0.35)	1.0 (0.86 to 1.0)	VERY LOW
Vriens 2018		0.45 (0.23 to 0.68)	0.97 (0.87 to 1.0)	VERY LOW

^a^QUADAS‐2 is a tool used for assessment of the quality of diagnostic accuracy studies. This tool comprises four domains: patient selection, index test, reference standard and flow and timing. Each domain is assessed in terms of risk of bias; the first three domains are also assessed in terms of concerns regarding applicability. CI: confidence interval DOR: diagnostic odds ratio PAD: peripheral arterial disease TBI: toe‐brachial index TSBP: toe systolic blood pressure

## Background

Peripheral arterial disease (PAD) is caused by atherosclerotic occlusive disease of the lower extremities in which the narrowing of arteries reduces blood flow to the lower limbs. This can eventually lead to total obstruction or occlusion of the arteries (Criqui 2015; Golledge 2022). This progressive stenosis of arterial beds impedes the delivery of essential nutrients to the tissues (Golledge 2022). PAD most commonly presents in the sixth and seventh decades of life. Prevalence is approximately 10% of people under 70 years of age and about 20% of people over 70 years of age (Golledge 2022; Hirsch 2001; Song 2019). The diagnosis of PAD is often overlooked in clinical practice, as over two‐thirds of PAD sufferers are either asymptomatic or present with symptoms that are atypical or non‐specific (Polonsky 2021). Common symptoms of PAD include distal pain, numbness and coldness. These non‐specific symptoms are often confused with other conditions, such as arthritis or nerve disorders (McDermott 2010). PAD can result in physical inactivity (due to muscle pain during exercise), limb ischaemia, non‐healing wounds, gangrene, limb amputation and death (Gerhard‐Herman 2016).

Clinicians from a variety of backgrounds rely on a range of invasive and non‐invasive diagnostic techniques to identify the presence and monitor the severity of lower extremity PAD. Commonly employed non‐invasive methods include the ankle‐brachial index, toe systolic blood pressure (TSBP), toe‐brachial index (TBI) (also known as toe‐brachial pressure index (TBPI)), Doppler velocity waveform analysis, pulse volume recording and colour duplex ultrasound (CDU) (Aboyans 2018; Conte 2019). Invasive methods of PAD diagnosis include contrast angiography, magnetic resonance angiography and computed tomography angiography (Song 2019).

The accuracy of the ankle‐brachial index is highly likely to be reduced by the presence of medial arterial calcification. Medial arterial calcification is a condition that stiffens the artery vessel wall (Smith 2008), preventing the compression of the artery by the cuff and sphygmomanometer. This results in a falsely elevated systolic ankle pressure measurement (Aboyans 2018). Populations that are most commonly affected by medial arterial calcification include people with diabetes or renal disease and those of advanced age (Towler 2008). However, the digital arteries are commonly spared from this condition, therefore the TBI is less likely to be affected (Aboyans 2018).

International guidelines recommend that people with clinical signs and symptoms of PAD, those considered clinically at risk due to co‐existing conditions including chronic kidney disease and diabetes, individuals over 50 years of age with a family history of PAD or smoking history, and individuals over 65 years have an annual assessment for PAD (Aboyans 2018; Fitridge 2023; Gerhard‐Herman 2016). This assessment involves taking a complete medical history, inspecting for clinical signs and symptoms of PAD, measuring blood pressure and palpating pulses (Aboyans 2018; Fitridge 2023; Gerhard‐Herman 2016). If features of PAD are identified, an ankle‐brachial index is recommended. However, in cases where the ankle‐brachial index is elevated beyond the normal range (i.e. > 1.4) secondary to medial arterial calcification, TSBP or the TBI is indicated (Aboyans 2018; Gerhard‐Herman 2016).

Many different health professionals utilise TSBP and the TBI in their practice, including nurses, podiatrists and physicians (Tehan 2015; Tehan 2019). TSBP and TBI can be measured using a variety of manual or automated techniques, including continuous wave Doppler, mercury strain‐gauge or photo plethysmography (Venermo 2012). The great toe or second toe can be used interchangeably to measure TSBP (Bhamidipaty 2015). Normal values for the TBI reported in the literature vary and include > 0.6, > 0.7 and > 0.75 (Hoyer 2013); there is no current evidence to determine which values are most commonly used in clinical practice. Some of the variation relates to whether the test is being used to establish the presence of PAD or predict the healing of a wound. A variety of thresholds for TSBP are reported in the literature as indicating pathology; however, these are frequently reported in the context of identifying severe PAD in the form of critical limb ischaemia or determining wound‐healing capacity (Linton 2020; Sonter 2014; Tay 2019). Inter and intra‐tester reliability of TSBP and TBI has been demonstrated to be acceptable; however, measurement error is broad (Pahlsson 2008; Romanos 2010; Sonter 2015). A diagnosis of PAD is generally based on a combination of signs and symptoms and an abnormally low result from an objective test, such as the ankle‐brachial index or TBI, or both.

A clinical diagnosis of PAD is managed with aggressive risk factor modification, including pharmacotherapy and lifestyle changes. Further imaging is used where revascularisation is being considered for either disabling symptoms or manifestations of limb‐threatening ischaemia (Golledge 2022).

### Target condition being diagnosed

Presence or absence of PAD in the lower limbs of adults 18 years of age and over.

### Index test(s)

TSBP and the TBI are the index tests. The TBI is the ratio between the systolic toe and brachial pressures and is calculated by dividing TSBP by the higher of the left and right brachial systolic pressures (Hoyer 2013). TSBPs are a measure of systolic blood flow in the great or second toe, whereby an occlusive pneumatic cuff is placed around the proximal phalanx and the return of pulsatility is measured with plethysmography, Doppler, laser Doppler or the strain‐gauge technique (Hoyer 2013). Brachial pressure is measured by taking systolic blood pressures at both arms (brachial arteries) in a supine position using a Doppler device, brachial cuff and sphygmomanometer (Gerhard‐Herman 2016; Norgren 2007).

Patients should be resting in a completely supine position, with the feet at the same level as the heart, for 10 minutes prior to measurement of both toe and brachial pressures (Sadler 2013). It is recommended that patients avoid caffeine intake, vigorous exercise and nicotine for two hours prior to pressure measurement. All of these factors interfere with toe and brachial pressure testing. The temperature of the room should be controlled to avoid it influencing skin temperature and affecting toe pressure measurement. Pre‐measurement warming of digits has been used in some cases to overcome issues with cool skin temperature in the periphery (Sawka 1992).

Currently, a range of values are used for interpretation of the TBI, with TBI values of < 0.6, < 0.7 and < 0.75 all being considered indicative of PAD (Hoyer 2013). For the purposes of this review, we will use < 0.7 as an indicator of PAD, as this threshold is used in current international guidelines (Gerhard‐Herman 2016; Norgren 2007). Interpretations of TSBP values also vary in the literature (Sonter 2014). International guidelines recommend a cut‐off of < 30 to < 50 mmHg as an indicator of severe limb ischaemia (severe PAD, advanced chronic limb‐threatening ischaemia (CLTI)) or wound‐healing capacity (Aboyans 2018; Gerhard‐Herman 2016; Norgren 2007; Zhan 2015). A previous study of the accuracy of TSBP for the diagnosis of PAD suggests a threshold of 96 mmHg in the general population (Tehan 2017).

### Clinical pathway

Management of risk factors following a diagnosis of PAD is used to reduce associated cerebrovascular and cardiovascular events and lower limb complications, including foot ulceration and amputation (Gerhard‐Herman 2016). Clinicians may use numerous different methods to detect PAD in practice, and current evidence suggests that what clinicians do is sometimes inconsistent with guidelines (Tehan 2015; Tehan 2019). Assessing for PAD entails taking a complete medical history, looking for signs and symptoms of PAD, pulse palpation and, if indicated, performing non‐invasive vascular diagnostic tests (Aboyans 2018; Fitridge 2023; Gerhard‐Herman 2016). Health care providers use the TBI to identify PAD in conjunction with other diagnostic testing methods, such as the ankle‐brachial index (ABI). The TBI is most frequently performed in populations in which medial arterial calcification is suspected or in the event of an elevated ABI result (Gerhard‐Herman 2016). TSBPs are also used in combination with other vascular testing methods, such as the ABI. TSBPs are used to identify the presence of PAD, often in a more severe form, and to predict wound‐healing capacity in patients with lower extremity wounds (Forsythe 2019).

Based on the results of these non‐invasive tests, patients are triaged into ongoing monitoring or referred for further intermediate testing, such as colour duplex ultrasound (CDU) and, if required, aggressive risk factor modification. Endovascular, hybrid or open surgical revascularisation techniques may be used when there is a negative impact on the quality of life or if the limb is at risk of amputation (Gerhard‐Herman 2016).

#### Prior test(s)

In patients suspected to have PAD, a thorough history and physical examination is recommended (Gerhard‐Herman 2016). History taking is focused on eliciting whether symptoms of claudication, impaired walking function or ischaemic rest pain are present. Physical examination consists of lower extremity pulse examination, auscultation of vascular bruits, observation of any non‐healing wounds, gangrene or other physical findings such as elevation pallor or dependent rubor. Further cursory tests may include capillary refill, changes in skin temperature and colour, hair loss or atrophy (Vriens 2018). The most commonly recommended initial bedside testing method in patients with suspected PAD is the ABI (Gerhard‐Herman 2016).

#### Role of index test(s)

Health care providers use the TBI to identify PAD in addition to other diagnostic testing methods, such as the ABI. The TBI is most frequently performed in populations in which medial arterial calcification is suspected or in the event of an elevated ABI result, or where an ABI is contra‐indicated. TSBPs are also used in combination with other vascular testing methods, such as the ABI. TSBPs are used to identify the presence of PAD, often in a more severe form, and to predict wound‐healing capacity in patients with lower extremity wounds.

#### Alternative test(s)

Alternative tests include the ABI, laser Doppler, skin perfusion pressure (SPP), Doppler velocity waveform analysis, pulse volume recording and transcutaneous oximetry (TCPO2). The diagnostic accuracy of alternative tests is not a subject of this review.

### Rationale

Accurate diagnosis of PAD is essential to effectively manage the disease and to prevent associated morbidity and mortality. TSBP and TBI measurements are simple, inexpensive and low‐risk diagnostic tests for PAD that can be performed by a broad range of health care providers in various settings. International guidelines currently recommend the TBI for the diagnosis of PAD when medial arterial calcification is suspected (Aboyans 2018; Gerhard‐Herman 2016), or in people with diabetes as part of routine clinical assessment (Fitridge 2023).

## Objectives

To (1) estimate the accuracy of TSBP and TBI for the diagnosis of PAD in the lower extremities at different cut‐off values for test positivity in populations at risk of PAD, and (2) compare the accuracy of TBI and TSBP for the diagnosis of PAD in the lower extremities.

### Secondary objectives

To investigate several possible sources of heterogeneity in test accuracy, including the following: patient group tested (people with type 1 or type 2 diabetes, people with renal disease and general population), type of equipment used, positivity threshold and type of reference standard.

## Methods

### Criteria for considering studies for this review

#### Types of studies

We included all diagnostic test accuracy studies using case‐control, cross‐sectional, prospective and retrospective designs in which all participants had either a TSBP or TBI measurement plus a validated method of vascular diagnostic imaging for PAD. We needed to be able to cross‐tabulate (2 x 2 table) the results of the index test and the reference standard to include a study. We also included any randomised diagnostic clinical trials using validated vascular diagnostic imaging that met these criteria.

#### Participants

All adults 18 years of age and over were eligible for inclusion. We included studies of symptomatic and asymptomatic participants. In some studies, TSBP or TBI is performed in the event of an elevated ABI. We included any of these studies, but did not combine them with studies of people receiving TBI as their initial assessment tool.

#### Index tests

TSBP and the TBI (also called toe‐brachial pressure index (TBPI)) are the index tests that are used in addition to other non‐invasive diagnostic tests when evaluating a patient with suspected PAD. We included data collected by photoplethysmography (PPG), laser Doppler, continuous wave Doppler, sphygmomanometers (both manual and aneroid) and manually operated or automated digital equipment. We also compared the diagnostic accuracy of TSBP and TBI.

#### Target conditions

Presence of PAD of the lower limbs.

#### Reference standards

We included studies that used as a reference standard the following diagnostic imaging methods for PAD: colour duplex ultrasound (CDU), digital subtraction angiography (DSA), magnetic resonance angiography (MRA) or multi‐detector computed tomography (MDCT). All of these testing methods are recommended to diagnose the location and severity of PAD (Conte 2019; Gerhard‐Herman 2016). Therefore, for the purpose of this review, these were deemed acceptable for use as a reference standard. We did not include ABI or a composite of non‐invasive tests for diagnosing PAD. The index test and reference standard should ideally be completed on the same day, but we deemed up to one month apart acceptable (Tschopl 1997). We included studies that did not report the duration between index and reference tests, and this is considered in the critical appraisal of methodology.

### Search methods for identification of studies

We did not apply any restrictions on date or language of publication or publication status of studies. We did not set any publication time limits. We did not use a search filter for the diagnostic method.

#### Electronic searches

The Cochrane Vascular Information Specialist searched the following databases for relevant studies:

- MEDLINE and Epub Ahead of Print, In‐Process & Other Non‐Indexed Citations and Daily (Ovid) (from 1946 onwards);
- Embase (Ovid) (from 1974 onwards);
- CINAHL (EBSCO) (from 1982 onwards);
- LILACS (BIREME) (from 1982 onwards);
- DARE (Database of Abstracts of Reviews of Effects) and HTA (the Health Technology Assessment Database) via crd.york.ac.uk/CRDWeb;
- ISI Conference Proceedings Citation Index ‐ Science via Web of Science;
- British Library Zetoc conference search via Zetoc Database.

The Information Specialist and review authors devised a draft search strategy for MEDLINE, which is displayed in Appendix 1. This strategy was used as the basis for search strategies for the other databases listed.

The Information Specialist also searched the following trial databases for details of ongoing and unpublished studies:

- ICTRP (WHO International Clinical Trials Registry Platform) (apps.who.int/trialsearch/);
- ClinicalTrials.gov (clinicaltrials.gov/).

The most recent searches were carried out on 27 February 2024.

#### Searching other resources

We also manually searched the reference lists and citations of included studies recursively for relevant studies. We contacted all authors of included studies and known researchers in the field in search of unpublished literature.

### Data collection and analysis

#### Selection of studies

Two review authors and a research assistant (PT, VC and MH), working independently, screened titles and abstracts retrieved by the electronic searches. One review author managed any conflicts (JM). Two review authors (VC and PT) independently screened the full‐text papers of potentially eligible studies. We resolved disagreements by discussion or, when needed, by mediation with another review author (JM or BP). Duplicates were removed by Covidence, with additional duplicates identified manually by one author (PT).

#### Data extraction and management

A standardised data extraction form was derived using RedCap data software and pilot tested (PT and BP). Four review authors (PT, VC, BP, SL), working independently, used this form to extract data from all included studies. Any conflicts were resolved by another review author (SL and BP).

All included studies reported results in a manner such that a 2 x 2 contingency table could be populated with the number of true positives (TP), false positives (FP), false negatives (FN) and true negatives (TN). In the instance that these values were not directly reported within the study, we calculated values from the reported sensitivity and specificity of the index test in conjunction with the total number of limbs and observed prevalence of PAD within the study. If more than one threshold for the index test was reported in a study, a single threshold was utilised for generation of the contingency table. For consistency of approach, we populated a single 2 x 2 contingency table containing the full group of study participants for inclusion in the development of forest plots and potential meta‐analysis. If results specific to a patient subgroup were reported, we also populated a 2 x 2 contingency table specific to the subgroup for potential use in a sensitivity analysis.

We also recorded the threshold(s) used for interpreting results, study design, study population, total number of participants, total number of limbs, prevalence of PAD, index test used, method of conduct of the index test (automated or manual), device or technique used for the index test, and the reference standard.

One full‐text paper required translation as it was published in Czech. We used Google Translate for this purpose.

#### Assessment of methodological quality

We used Quality Assessment of Diagnostic Accuracy Studies‐2 (QUADAS‐2) (Whiting 2011) to develop a quality assessment tool. QUADAS‐2 has four domains: patient selection, index test, reference standard and flow and timing. Each domain is assessed in terms of risk of bias. The first three domains also include assessment of applicability. Review‐specific signalling questions and appropriate items concerning the applicability of primary studies relative to the review, together with guidance about rating, can be found in Appendix 2. Additional questions used in QUADAS‐2 for this review are the following:

- TBI or TSBP: Was the index test measured using an adequately detailed, appropriately described and executed testing methodology?
- Where both TSBP and TBI measurements were taken, were the index tests interpreted blinded to each other?

Two review authors completed the quality appraisal (PT and BP), working independently, with a third review author resolving any disagreements (SL). The third review author also completed the quality appraisal for included studies published by one review author (PT) to avoid any conflicts of interest or bias.

#### Statistical analysis and data synthesis

##### Objective 1

We considered the true disease status as a binary variable. We used 2 × 2 contingency tables populated with participant‐level data (numbers of TPs, TNs, FPs and FNs). We intended to only pool studies in the meta‐analysis if participant groups were considered to be clinically similar. Since there was likely to be variability in thresholds used across studies, we planned to pool the summary receiver operating characteristic (ROC) curve using the hierarchical summary receiver operating characteristic (HSROC) model proposed by Rutter and Gatsonis (Rutter 2001). We intended to estimate the parameters from this model using the NLMIXED procedure in SAS software version 9.4 (SAS 2014).

##### Objective 2

We intended to compare the diagnostic accuracy between the two testing methods (TSBP and TBI) by including method type as a binary covariate in the HSROC model. We planned to include all studies in this analysis (indirect comparison), not just studies that used both tests.

We made forest and scatter plots of individual study estimates of sensitivity and specificity for each index test as preliminary investigations of the presence of between‐study variation in test accuracy.

We intended to complete the meta‐analyses using the NLMIXED procedure in SAS v9.4. We produced forest plots using Review Manager 5.4 (RevMan) (RevMan 2020).

#### Investigations of heterogeneity

Heterogeneity was to be assessed graphically by highlighting departures of individual study estimates from the summary ROC curve as well as departures between 95% prediction and confidence regions. We planned to include possible sources of heterogeneity as covariates in the HSROC model to explore their effect on heterogeneity in test positivity, position and shape of the summary ROC curve. We also intended to present estimates of the random‐effects variance (in the log odds scale). If there appeared to be significant departures and there was a reasonable number of sites contributing data (e.g. > 10), we planned to include sources of heterogeneity as covariates in the bivariate regression model to explore their effect on heterogeneity. This was to include patient group tested (people with type 1 or type 2 diabetes, people with renal disease and the general population), type of equipment used (photoplethysmography, laser Doppler, continuous wave Doppler, sphygmomanometers (both manual and aneroid) and by manually operated or automated digital equipment), index (TSBP and TBI) and type of reference standard (CDU, DSA, MRA, MDCT). We planned to group other factors identified as potential sources of heterogeneity in ROC plots for visual assessment for heterogeneity. As we were to base these assessments on study‐specific data (rather than participant‐specific data), we planned to interpret results cautiously. We planned to use likelihood ratio tests to assess the effect of adding or removing variables from the regression model, together with assessing reductions in Akaike information criterion and Bayesian information criterion.

#### Sensitivity analyses

If sufficient data were available from the included studies and limited heterogeneity was present, we intended to complete sensitivity analyses to explore the effect of risk of bias and study characteristics on the accuracy of TSBP and the TBI. Inclusion in the meta‐analyses was limited to:

- studies that used a cross‐sectional study design (we removed case‐control studies);
- studies that used manual measurement;
- studies that used automated measurement.

Additional sensitivity analyses were also planned, limiting inclusion in the meta‐analyses to:

- studies that used CDU as the reference standard;
- studies that used a patient population of people with type 1 or type 2 diabetes (for studies where the patient group included the general population with results also reported specific to a diabetes subgroup, we included the 2 x 2 contingency table specific to this subgroup).

#### Assessment of reporting bias

Results were to be interpreted cautiously and in the context of possible sources of publication bias. We planned to use a funnel plot using log(DOR) against 1/sqrt(effective sample size) to assess reporting bias (Deeks 2005).

#### Assessment of the certainty of the evidence

We used the GRADE approach for diagnostic studies to determine the certainty of the evidence (Balshem 2011; Schünemann 2008; Schünemann 2016). We rated the certainty of the evidence as high, moderate, low or very low. We planned to base this decision on assessment using four domains of the GRADE system as follows (GRADEpro GDT; Schünemann 2020a; Schünemann 2020b). Two authors (PT and BP), working independently, completed the GRADE assessment with any disagreements resolved by discussion or by a third author (VC).

- Risk of bias ‐ using the QUADAS‐2 tool.
- Indirectness ‐ using the QUADAS‐2 tool to assess applicability concerns and look for important differences between the populations studied.
- Inconsistency ‐ explored in accordance with GRADE by downgrading for unexplained inconsistency in sensitivity and specificity estimates. We carried out prespecified analyses to investigate potential sources of heterogeneity and downgraded when we believed we could not explain the inconsistency in the accuracy estimates.
- Imprecision ‐ we examined the length of the confidence intervals and asked whether the truth set at the lower or upper limit of the 95% confidence interval would change the decision. We intended to calculate projected ranges for TPs, TNs, FPs and FNs for a given prevalence of disease and make judgements on imprecision from these calculations.

We constructed a summary of findings table that presents the main review findings along with the certainty of the evidence.

## Results

### Results of the search

A total of 20,961 records were returned, with 15,283 titles and abstracts screened, and 140 full‐text articles assessed for inclusion. Eighteen studies (19 records) met the criteria and were included (Figure 1). We excluded 121 studies (121 records) with reasons (see Characteristics of excluded studies). A summary of the characteristics of each of the included studies is displayed in Table 2 and Characteristics of included studies.

**1:**
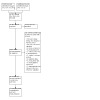
Study flow diagram

**1:** Description of included studies

**Study ID/study design/region**	**Index**	**Equipment/conduct/tester**	**Blinding** **Flow/timing**	**Threshold**	**Reference**	**Population/** **% diabetes**	**Mean age/female (%)**	**Participants (limbs)**	**PAD prevalence (%)**
AbuRahma 2020 Observational ‐ retrospective USA	TBI	Doppler Manual/accredited laboratory	‐/‐	0.70	CDU	General/62	65.4/‐	297 (297)	58.6
Babaei 2020 Observational ‐ prospective Iran	TBI	Doppler Manual/trained nurse	Blinded/same day	0.38	CDU	Diabetes/100	60.7/60.2	303 (597)	2.2
Chen 2021 Observational ‐ prospective USA	TBI	PPG Manual/trained research staff	‐/‐	0.70	CDU	Renal/49	69/35	100 (195)	16.4
Cleofort 2023 Observational ‐ prospective France	TBI	Doppler Manual/not stated	‐/‐	0.70	CDU	Hospitalised patients with leg or foot ulceration, aged over 70 years/0	83.38/64	50 (100)	76
Fejfarova 2021 Observational ‐ prospective Czech Republic	TBI TSBP	Doppler Manual/not stated	‐/‐	0.60 60	CDU	Diabetes/100	67.5/‐	107 (107)	56
Fendrik 2023 Observational ‐ prospective Hungary	TBI	Laser Doppler/PPG Automated	‐/‐	0.70	CDU/Angiography/CTA	General/35	63.2/58.1	117 (223)	26
Machaczka 2021 Observational ‐ prospective Czech Republic	TBI	PPG Manual/not stated	‐/‐	0.70	CDU	Diabetes/100	64/42	21 (42)	60
Normahani 2020 Observational ‐ prospective United Kingdom	TBI	PPG Manual/vascular scientists	Blinded/same day	0.75	CDU	Diabetes/100	73/32.5	305 (234)	60.3
Okamoto 2006 Observational ‐ prospective Japan	TBI	PPG Auto/not stated	‐/‐	0.60	MDCT	Renal/‐	61.9/‐	36 (72)	64
Park 2012 Observational ‐ prospective Korea	TBI	PPG Auto/trained vascular nurses	‐/same day	0.60	Angiography	Diabetes/100	‐/‐	15 (30)	43
Singhania 2024 Observational‐ prospective India	TBI	PPG Auto/researchers	‐/‐	0.6	CTA	Diabetes over 50 years old/100	59.1 (PAD) 55.1 (no PAD)/29	175 (175)	24
Sonter 2017 Observational ‐ prospective Australia	TBI TSBP	PPG Auto/podiatrists	‐/< 7 days	0.70	CDU	General/56	73/42	90 (90)	42
Tehan 2016 Observational ‐ prospective Australia	TBI	PPG Manual/vascular sonographers	Not blinded/same day	0.70	CDU	General/diabetes 62	72.5/37	117 (117)	46
Tehan 2017 Observational‐ retrospective Australia	TSBP	PPG Manual/vascular sonographer	‐/‐	97	CDU	General/44	74.6/35	394 (394)	71.1
Tehan 2021 Observational ‐ prospective Australia	TBI TSBP	PPG Manual/vascular sonographer	‐/same day	0.70 60	CDU	General/40	68/34	50 (50)	52
Tsuyuki 2013 Observational ‐ prospective Japan	TBI	Other Auto/not stated	‐/‐	0.60	CTA	Renal/‐	67.2/37	16 (32)	50
Vriens 2018 Observational ‐ prospective United Kingdom	TBI TSBP	Laser Auto/vascular surgery fellow	Blinded/‐	0.75 50	CDU	Diabetes/ 100	66/25	60 (60)	33
Williams 2005 Observational ‐ prospective Case/control United Kingdom	TBI	PPG Manual/examiners	Not blinded/same day	0.75	CDU	General/85	63/26	68 (122)	29

CDU: colour duplex ultrasound CTA: computed tomographic angiography MDCT: multi‐detector computed tomography PAD: peripheral arterial disease PPG: photoplethysmography TBI: toe‐brachial index TSBP: toe systolic blood pressure

Funding details of all studies are outlined in Table 3.

**2:** Reported funding sources

**Study**	**Funding statement**
AbuRahma 2020	‐
Babaei 2020	The author(s) received no financial support for the research, authorship, and/or publication of this article.
Chen 2021	This study was funded by the National Institute of General Medical Sciences of the National Institutes of Health P20GM109036, Bethesda, Maryland and Tulane University Bridge Fund, New Orleans, Louisiana. This study was supported by Tulane Centers of Biomedical Research Excellence for Clinical and Translational Research in cardiometabolic Diseases P20 GM109036 and Tulane University Bridge Fund. The funders had no role in the design and conduct of the study; collection, management, analysis, and interpretation of the data; preparation, review, or approval of the manuscript; and decision to submit the manuscript for publication.
Cleofort 2023	This work did not receive any grant from funding agencies in the public, commercial, or not‐for‐profit sectors.
Fejfarova 2021	This study was supported by the Ministry of Health of the Czech Republic through grant NU20‐01‐00078 and its conceptual development of research organizations programme (Institute for Clinical and Experimental Medicine – IKEM, IN00023001).
Fendrik 2023	This research received no external funding.
Machaczka 2021	The publication was created with the support of a grant with registration number SGS07/LF/2019: "Evaluation of the validity of the toe brachial index in diabetics" within the Student Grant Competition of the University of Ostrava.
Normahani 2020	We acknowledge generous funding from Chelsea and Westminster Plus charity. During the planning and initial recruitment phase of this project Pasha Normahani was funded by a National Institute of Health Research (NIHR) Academic Clinical Fellowship.
Okamoto 2006	“Support: none”
Park 2012	‐
Singhania 2024	The study was funded partly by the Endocrine Society of India, and Research Society for Study of Diabetes in India (RSSDI), West Bengal chapter, India.
Sonter 2017	The financial assistance for this study was provided by a Ramaciotti foundation grant.
Tehan 2016	This project was funded through a University of Newcastle Faculty of Health Pilot Grant and a Hunter Medical Research Institute pilot grant.
Tehan 2017	This project was funded through a University of Newcastle new staff grant (G1100272) and early career researcher grant (G1300869).
Tehan 2021	The authors received no financial support for the research, authorship, and/or publication of this article.
Tsuyuki 2013	‐
Vriens 2018;	Funding sources: none
Williams 2005	Financial aid was provided by departmental funds.

The studies included 2321 participants with studies using one or both limbs of the participant in the assessment of the index test. A total of 3023 limbs were included with 1139 (mean 44.4%) affected by PAD. The prevalence of PAD ranged from 2.2% to 71.1.0%.

Of the 18 studies, 13 evaluated the TBI only (AbuRahma 2020; Babaei 2020; Cleofort 2023; Chen 2021; Fendrik 2023; Machaczka 2021; Normahani 2020; Okamoto 2006; Park 2012; Singhania 2024; Tehan 2016; Tsuyuki 2013; Williams 2005), one evaluated TSBP only (Tehan 2017), and four evaluated both the TBI and TSBP (Fejfarova 2021; Sonter 2017; Tehan 2021; Vriens 2018). The included studies used a range of different diagnostic thresholds (Table 2). Two studies reported more than one threshold for the TBI (Fejfarova 2021; Sonter 2017).

Seven studies included only participants with diabetes (Babaei 2020; Fejfarova 2021; Machaczka 2021; Normahani 2020; Park 2012; Singhania 2024; Vriens 2018), three studies included only participants with renal disease (Chen 2021; Okamoto 2006; Tsuyuki 2013), two studies included participants from the general population (Fendrik 2023; Tehan 2021), four studies included participants from the general population with data also reported specific to the participants with diabetes (Sonter 2017; Tehan 2016; Tehan 2017; Williams 2005), one study included participants from the general population with data also reported specific to the participants with diabetes and renal disease (AbuRahma 2020), and one study included hospital inpatients with active leg or foot ulceration (Cleofort 2023).

Twelve studies utilised PPG in the conduct of the index test (Chen 2021; Fendrik 2023; Machaczka 2021; Normahani 2020; Okamoto 2006; Park 2012; Singhania 2024; Sonter 2017; Tehan 2016; Tehan 2017; Tehan 2021; Williams 2005), four studies used continuous wave Doppler (AbuRahma 2020; Babaei 2020; Cleofort 2023; Fejfarova 2021), two studies used laser Doppler (Fendrik 2023; Vriens 2018), and two studies reported using an oscillometric method (Fendrik 2023; Tsuyuki 2013). Eleven studies used a manual measurement (AbuRahma 2020; Babaei 2020; Cleofort 2023; Chen 2021; Fejfarova 2021; Machaczka 2021; Normahani 2020; Tehan 2016; Tehan 2017; Tehan 2021; Williams 2005), and seven studies used an automated measurement (Fendrik 2023; Okamoto 2006; Park 2012; Singhania 2024; Sonter 2017; Tsuyuki 2013; Vriens 2018).

A reference standard of CDU was used in 13 studies (AbuRahma 2020; Babaei 2020; Chen 2021; Cleofort 2023; Fejfarova 2021; Machaczka 2021; Normahani 2020; Sonter 2017; Tehan 2016; Tehan 2017; Tehan 2021; Vriens 2018; Williams 2005), two studies used computed tomography angiography (angio CT) (Park 2012; Tsuyuki 2013), one study used multi‐detector computed tomography (MDCT) (Okamoto 2006), one study used CTA (Singhania 2024), and one study used any of three available reference standards (angiography, CTA, CDU).

We contacted the authors of potentially eligible papers, requesting either clarification on the performance and interpretation of the reference standard, or for the provision of information required to populate the 2 x 2 contingency tables. We contacted five authors on 25 March 2022, with two responding with clarifications and three not responding. We contacted four authors on 4 April 2024, with two responding with data for 2 x 2 contingency tables and two not responding.

### Methodological quality of included studies

We appraised the methodological quality of the included studies using the QUADAS‐2 instrument (Figure 2; Figure 3). Overall, the quality was low with an uncertain risk of bias in most domains. The main areas of concern related to inadequate reporting of patient selection (sampling) methods, blinding of the tester to the results of the index and/or reference standard, and reporting of timing between the performance of the index test and the reference standard.

**2:**
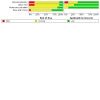
Risk of bias and applicability concerns graph: review authors' judgements about each domain presented as percentages across included studies

**3:**
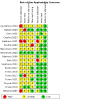
Risk of bias and applicability concerns summary: review authors' judgements about each domain for each included study

Three studies demonstrated high risk of bias for the patient selection domain (AbuRahma 2020; Fejfarova 2021; Williams 2005). One study used screening criteria to enrol participants who had transcutaneous oxygen measures between 30 and 50 mmHg at rest only (Fejfarova 2021), therefore inappropriately excluding potential participants, one study excluded people without symptoms (AbuRahma 2020), and one utilised a case‐control design and excluded people with signs or symptoms of severe disease (Williams 2005). Three studies had a high risk of bias for the index test, with two not pre‐specifying a diagnostic threshold, but rather using a data‐driven approach (Babaei 2020; Tehan 2017) and another study presenting a range of results based on differing thresholds (Fejfarova 2021). All of the included studies were either at low risk or unclear risk of bias for the reference standard domain.

In relation to flow and timing, 11 studies had an unclear risk of bias. Ten of the studies had inadequate reporting of the time between index and reference standard testing (AbuRahma 2020; Chen 2021; Cleofort 2023; Fejfarova 2021; Machaczka 2021; Okamoto 2006; Singhania 2024; Tehan 2017; Tsuyuki 2013; Vriens 2018), and for one study it was unclear if all patients received the same reference standard (Park 2012). Another study was at high risk of bias due to the use of three different reference standards and unclear timing between index and reference standard testing (Fendrik 2023).

Two studies had a high risk for applicability of patient selection (Fejfarova 2021; Park 2012), with one study recruiting participants with severe disease only (claudication or gangrene) (Park 2012), and another study including participants who had results within a pre‐specified range of another vascular test (Fejfarova 2021). Five studies had unclear risk for applicability of patient selection, with one study not including participants undergoing dialysis (Chen 2021), one study including controls with no risk factors, signs or symptoms of vascular disease (Fendrik 2023), one study not including participants with vascular disease symptoms (AbuRahma 2020), one study excluding participants with active wounds (Machaczka 2021), and one study excluding participants with both wounds or symptoms of vascular disease (Williams 2005). Five studies had unclear risk for applicability of the index test (AbuRahma 2020; Cleofort 2023; Fejfarova 2021; Okamoto 2006; Park 2012). This was due to inadequate description of test conduct and conditions. One study had a low risk of bias across all domains (Normahani 2020); this was conducted in a sample of 305 participants with diabetes. Most of the included studies lacked detail on the qualifications and experience of the tester, and whilst most of the included studies used CDU as a reference standard there was variation in the interpretation of the CDU results and the classification of PAD. In other reference standards, there was a similar lack of description of the interpretation of the reference standard to diagnose PAD.

We completed an assessment of GRADE certainty of evidence at individual study level. We were not able to make an overall judgement of the certainty of the evidence from pooled data due to the inability to conduct a meta‐analysis. The GRADE certainty of evidence assessment for individual studies was generally very low (Table 1). This downgrading was due to the results of the risk of bias assessment, inconsistency across included studies and imprecision within the included studies. The majority of the included studies had small sample sizes and there was a high level of imprecision with very wide confidence intervals noted for most studies.

### Findings

We could not complete meta‐analyses and sensitivity analyses due to high levels of heterogeneity across the included studies for both TBI and TSBP. In place of a meta‐analysis, we have provided a narrative synthesis and created forest and scatter plots to visually describe the findings of the included studies (Figure 4; Figure 5).

**4:**
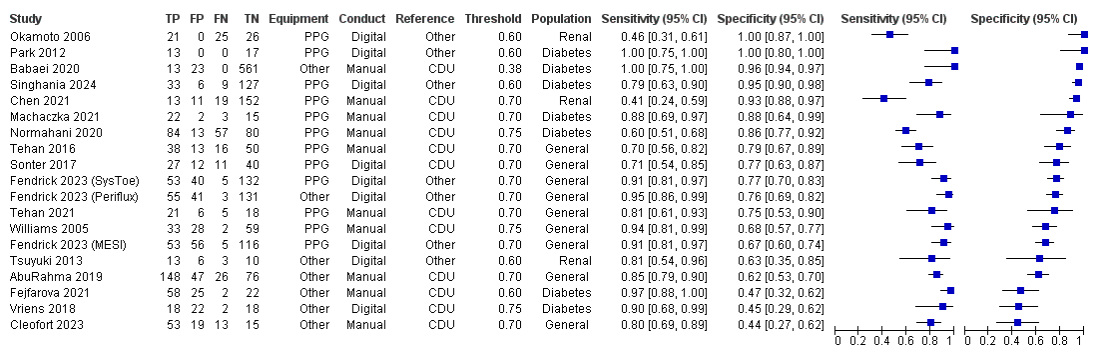
Forest plot of toe‐brachial index

**5:**
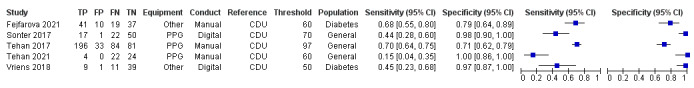
Forest plot of toe systolic blood pressures

#### Test accuracy TBI

The TBI was investigated in 17 studies that included 1927 participants, of which 2550 limbs had the TBI measured. For studies in which the TBI was investigated, an average of 44 out of every 100 (44.3%) limbs were found to have PAD, with this number ranging from 2 out of every 100 (2.2%) to 66 out of every 100 (66.0%). A high degree of heterogeneity can be observed in the study estimates for both sensitivity and specificity (Figure 4; Figure 6). The range of values for sensitivity and specificity was similar, with sensitivity estimates ranging from 0.41 to 1.00 and specificity estimates ranging from 0.44 to 1.00 (Figure 4).

**6:**
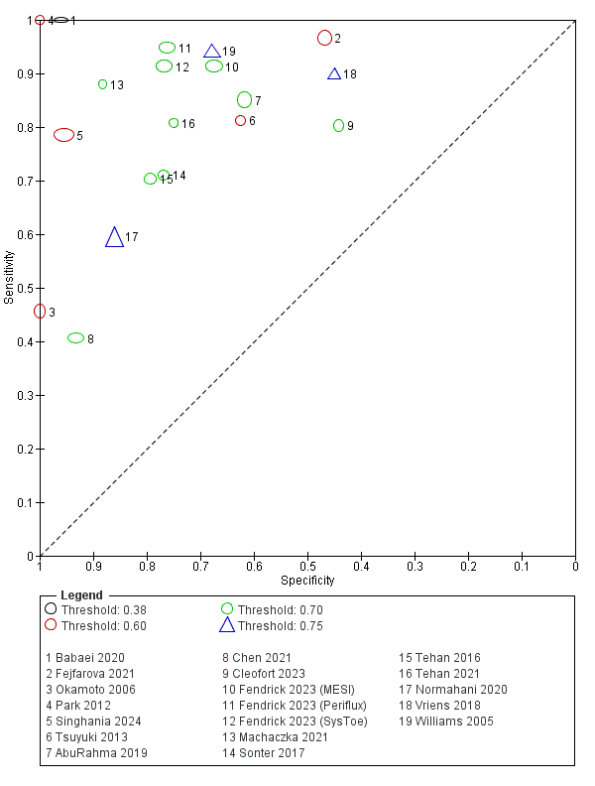
TBI plot in SROC space with thresholds

Four different thresholds were used across the 17 studies (Table 2): one used a threshold of 0.38 (Babaei 2020), five used a threshold of 0.60 (Fejfarova 2021; Okamoto 2006; Park 2012; Singhania 2024; Tsuyuki 2013), eight used a threshold of 0.70 (AbuRahma 2020; Cleofort 2023; Fendrik 2023; Fejfarova 2021; Machaczka 2021; Sonter 2017; Tehan 2016; Tehan 2021), and three used a threshold of 0.75 (Normahani 2020; Vriens 2018; Williams 2005).

#### Test accuracy TSBP

Toe systolic blood pressure was investigated in five studies that included 701 participants, of which 701 limbs had TSBP measured. In the small number of studies available, some heterogeneity can be observed (Figure 5; Figure 7). For studies in which TSBP was investigated, an average of 51 out of every 100 (51.2%) limbs were found to have PAD, with this number ranging from 33 out of every 100 (33.3%) to 71 out of every 100 (71.1%). In the small number of studies available, large amounts of heterogeneity can be observed, particularly in sensitivity values. The range of values for sensitivity was 0.15 to 0.70 with specificity estimates ranging from 0.71 to 1.00. Of the five studies evaluating TSBP, four different thresholds were used, with these being 50 mmHg (Vriens 2018), 60 mmHg (Fejfarova 2021), 70 mmHg (Sonter 2017), and 96 mmHg (Tehan 2017), respectively.

**7:**
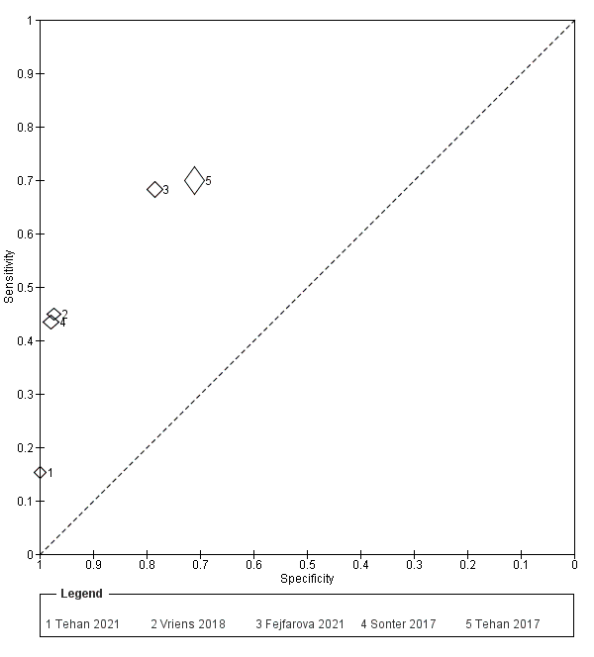
TSBP plot in SROC space with thresholds

#### Secondary objectives

##### For comparative accuracy of TBI and TSBP (indirect comparison of all studies)

Whilst 18 studies allowed for indirect comparison between the accuracy of TBI and TSBP, we did not perform statistical comparisons due to heterogeneity.

##### Direct comparison of TBI and TSBP (restricted to comparative studies)

Direct comparison of the two index tests was performed in only four studies (Fejfarova 2021; Sonter 2017; Tehan 2021; Vriens 2018). We did not perform any meta‐analysis due to the small number of studies.

##### Investigations of heterogeneity

Statistical analysis of heterogeneity was planned, to investigate the contribution of different factors, such as patient populations, thresholds for index tests, differing reference standards and methods of measurement (manual versus automated). Due to the small number of included studies and the amount of heterogeneity, this could not be performed. The main identified sources of heterogeneity were: varied participant groups including varying levels of PAD prevalence, sampling methods, varying thresholds used for index test, different types of measurement, methods of measurement and varying equipment types (manual versus automated), inadequate detail on testers, different reference standards and unclear interpretation of reference standards.

#### Sensitivity analysis

This could not be completed due to insufficient data and heterogeneity across studies.

#### Assessment of reporting bias

Due to high levels of heterogeneity, we were not able to use a funnel plot to visually assess reporting bias. However, due to the variation in results across the included studies, we deemed the risk of reporting bias to be low.

#### Assessment of the certainty of evidence

We were unable to determine the certainty of evidence from pooled studies, due to the inability to perform a meta‐analysis. We used GRADE to assess the certainty of evidence at individual study level (Table 1). The majority of studies had either low or very low certainty of evidence.

## Discussion

This review aimed to assess the accuracy of TSBP and TBI for diagnosing PAD in populations at risk of the condition. Secondary aims of this review were to assess the comparative accuracy of TBI and TSBP indirectly and directly. Eighteen studies met the eligibility criteria (Table 1). Studies were generally of low methodological quality, with low to very low certainty of evidence.

- Seventeen studies examined the diagnostic test accuracy of TBI, with sensitivity ranging from 0.41 to 1.00 and specificity from 0.44 to 1.00.
- Five studies examined the diagnostic test accuracy of TSBP, with sensitivity ranging from 0.15 to 0.70 and specificity from 0.71 to 1.00.

Due to the heterogeneity of the included studies, including methodological differences and differences in diagnostic thresholds and population characteristics, as well as the use of a data‐driven diagnostic threshold or multiple thresholds, we were unable to pool the results of the studies. This prevented formal evaluation and comparison of the diagnostic test accuracy of TBI and TSBP for PAD.

In accordance with GRADE, the certainty of evidence was low or very low in all studies. We generally downgraded certainty due to the combination of unclear or high risk of bias in quality assessment results, inconsistency across sensitivity and specific estimates between studies, and imprecision in multiple studies with wide confidence intervals around sensitivity and specificity estimates. Inconsistency and imprecision were particularly evident in the studies evaluating TSBP.

For risk of bias, there was a lack of clarity in the reporting of several items across the studies, including items such as sampling methods, blinding between the index and reference standard, information relating to the tester and information relating to the timing between the index and reference standard. There were also some applicability concerns in a minority of studies that included samples which may not be representative of the population who would require the test to be conducted, with either very high prevalence of severe PAD or very low prevalence of PAD. All of the included studies used valid vascular imaging as a reference standard; however, there was some variation and a lack of reporting on the interpretation of some of these methods that may impact applicability. Furthermore, there was a lack of reporting on the training and experience of the CDU operator. This is of concern, given that the test is operator dependent.

The heterogeneity of the included studies was significant, precluding meta‐analysis or sensitivity analysis. This is partially a result of our broad inclusion criteria; however, the lack of capacity for sub‐analysis to be conducted highlights the few available diagnostic test accuracy studies for TBI and TSBP. The prevalence of PAD was also extremely varied, ranging from 2% to 76% of the study population. Clinical variability between populations, for example, a patient population with more severe disease, may result in a test having greater or lesser diagnostic accuracy (Leeflang 2009). Furthermore, aspects of study design, including patient sampling, equipment and testing methods, and different reference standards may have influenced both disease prevalence and test accuracy. One of the greatest sources of heterogeneity in the included studies was related to the study populations. Three studies included people with renal disease, a further seven studies were restricted to those with diabetes and eight studies included people with and without diabetes. Renal disease and diabetes are associated with increased risk of medial artery calcification and more distally distributed atherosclerotic disease patterns, both of which can affect the accuracy of reference and index tests included in this review (Golledge 2022; Jude 2001; Low Wang 2018).

Seven of the included studies were in individuals with diabetes only, whereas another eight studies included varying proportions of diabetes participants in their sample. It is well established that diabetes populations more commonly experience multi‐segment PAD, with more frequent long‐segment occlusions and disease that predominantly affects infra‐popliteal arteries. Furthermore, medial arterial calcification is more commonly present in individuals with diabetes, and affects the accuracy of non‐invasive testing methods, including TSBP (Ix 2012). Additionally, three studies were in renal failure populations, who also experience more prevalent and severe infra‐popliteal disease, with a high incidence of medial arterial calcification (Baghdasaryan 2020; O'Hare 2001). These factors will influence the performance of TBI and TSBP, as well as commonly used reference standards, making the results of these studies less certain.

This study is the first Cochrane review on TBI and TSBP for the diagnosis of PAD. The eligibility criteria for this review were inclusive of all diagnostic accuracy studies using different methodologies in any adult population with or without symptoms of PAD. These broad criteria led to the inclusion of 18 studies, however they have resulted in issues with heterogeneity. This differs from a previously published Cochrane review on the ankle‐brachial index for the diagnosis of lower limb PAD, which included studies in symptomatic PAD patients only (Crawford 2016). This narrower focus avoided issues with heterogeneity, however it resulted in the review only including one study that met their criteria. This further highlights the lack of research in non‐invasive vascular assessment of the lower limb.

### Summary of main results

TSBP and TBI are non‐invasive tests used to identify PAD and they have the potential to be clinically useful. However, the results of this review have demonstrated that there are only a small number of studies available. These studies are generally of unclear methodological quality, mostly providing low to very low certainty of evidence, and there is a large amount of heterogeneity between them. The sensitivity and specificity of TSBP and TBI across the included studies was varied, and the low or very low certainty of evidence makes the findings even less certain. There is therefore currently inadequate evidence to support the use of these tests in clinical practice.

### Strengths and weaknesses of the review

The strengths of this review include the in‐depth and comprehensive search of the literature, including double screening and extraction by review authors who are both clinicians and researchers. The application of methodological quality appraisal was done in a transparent and reproducible manner. In addition, this is the first systematic review to examine the diagnostic accuracy of TSBP and TBI across mixed populations.

The weakness of the review was the small number of studies available that met the inclusion criteria, particularly for TSBP. This review excluded 121 studies, the majority of which were either not diagnostic test accuracy studies (n = 69) or utilised the wrong reference standard (n = 28). This highlights the lack of available studies. The small number of studies, as well as the high level of heterogeneity, led to our inability to complete a meta‐analysis, meaning that it is not possible to draw robust conclusions. According to our protocol, studies that did not describe the timing between the index and reference standard were included, whereas we excluded studies where this was reported as more than one month. This is a limitation and future iterations of this review should consider clarifying this specific exclusion criterion.

### Applicability of findings to the review question

All the included studies were studies of diagnostic test accuracy. However, not all of the studies evaluated TSBP or the TBI as the primary outcome. Therefore, there was some indirectness in a minority of studies. The populations studied within this review were broad, defined as populations at risk of PAD. This created issues with heterogeneity. Similarly, we did not place restrictions on setting, however most studies were conducted in either primary or secondary care settings. There was also variety in the equipment type utilised. TSBP and the TBI were conducted similarly across the included studies, however some studies did not report the methods in sufficient detail to allow replication.

## Authors' conclusions

**Implications for practice**

Both toe‐brachial index (TBI) and toe systolic blood pressure (TSBP) are accessible, low‐cost bedside tests that can be readily applied in practice. Due to the very low certainty of evidence and the small number of included studies, there is currently limited evidence in this Cochrane review to support TBI and TSBP as adjunct testing methods. Clinicians should therefore use caution when interpreting these tests in clinical practice.

**Implications for research**

There is a lack of high‐quality evidence to determine the diagnostic test accuracy of the TBI and TSBP for the diagnosis of peripheral arterial disease (PAD). More prospective, well‐designed studies with larger sample sizes are required. These studies should include representative samples who are recruited in a consecutive or random fashion, include appropriate blinding of testers to the index and/or reference standard, and be conducted in populations that include a spectrum of PAD prevalence. More comparative head‐to‐head studies of TBI and TSBP are also needed.

## History

Protocol first published: Issue 11, 2020

## Appendices

### Appendix 1. Sources searched and search strategies

**Table:**

**Source**	**Search strategy**	**Hits retrieved**
1. Ovid MEDLINE Epub Ahead of Print, In‐Process & Other Non‐Indexed Citations, Ovid MEDLINE Daily and Ovid MEDLINE 1946 to present (Date of most recent search: 26 February 2024	1 Ankle Brachial Index/ 2 (toe* adj4 brachial).ti,ab,kf. 3 (toe* adj4 (index or indices)).ti,ab,kf. 4 (toe* adj4 pressure).ti,ab,kf. 5 exp Toes/ and systol*.ti,ab,kf. 6 exp Toes/ and plethysmography/ 7 exp Toes/ and blood pressure/ 8 exp Toes/ and arterial pressure/ 9 exp Toes/ and bp.ti,ab,kf. 10 exp Toes/ and brachial artery/ 11 exp Toes/ and regional blood flow/ 12 exp Toes/ and blood flow velocity/ 13 exp Toes/ and Ultrasonography, Doppler, Duplex/ 14 TBI.ti,ab,kf. 15 TBPI.ti,ab,kf. 16 TAI.ti,ab,kf. 17 (hallux adj4 brachial).ti,ab,kf. 18 (hallux adj4 pressure).ti,ab,kf. 19 (hallux adj4 index).ti,ab,kf. 20 photoplethysmograph*.ti,ab,kf. 21 or/1‐20 22 exp Peripheral Vascular Diseases/di 23 Arterial Occlusive Diseases/di 24 exp Arteriosclerosis/di 25 exp Peripheral Arterial Disease/di 26 Intermittent Claudication/di 27 (atherosclero* or arteriosclero* or PVD or PAOD or PAD).ti,ab,kf. 28 (arter* adj4 (occlus* or steno* or obstuct* or lesio* or block* or obliter*)).ti,ab,kf. 29 (vascular adj4 (occlus* or steno* or obstuct* or lesio* or block* or obliter* or califi*)).ti,ab,kf. 30 (peripher* adj4 (occlus* or steno* or obstuct* or lesio* or block* or obliter*)).ti,ab,kf. 31 (peripheral adj3 disease*).ti,ab,kf. 32 arteriopathic.ti,ab,kf. 33 (claudic* or hinken*).ti,ab,kf. 34 (critical limb ischemia or CLI).ti,ab,kf. 35 dysvascular*.ti,ab,kf. 36 ((leg or legs) adj4 (obstruct* or occlus* or stenos* or block* or obliter*)).ti,ab,kf. 37 ((limb or limbs) adj4 (obstruct* or occlus* or stenos* or block* or obliter*)).ti,ab,kf. 38 (lower adj3 extremit* adj4 (obstruct* or occlus* or stenos* or block* or obliter*)).ti,ab,kf. 39 exp Atherosclerosis/di 40 or/22‐39 41 21 and 40 42 exp animals/ not humans.sh. 43 41 not 42	Dec 2020: 3028 Aug 2021: 348 Aug 2022: 368 February 2024: 212
2. Embase via OVID 1972‐2021 (Date of most recent search: 26 Feburary 2024)	1 exp ankle brachial index/ 2 (toe* adj4 brachial).ti,ab. 3 (toe* adj4 (index or indices)).ti,ab. 4 (toe* adj4 pressure).ti,ab. 5 exp Toes/ and systol*.ti,ab. 6 exp Toes/ and plethysmography/ 7 exp Toes/ and blood pressure/ 8 exp Toes/ and arterial pressure/ 9 exp Toes/ and bp.ti,ab. 10 exp Toes/ and brachial artery/ 11 exp Toes/ and regional blood flow/ 12 exp Toes/ and blood flow velocity/ 13 exp Toes/ and Ultrasonography, Doppler, Duplex/ 14 TBI.ti,ab. 15 TBPI.ti,ab. 16 TAI.ti,ab. 17 (hallux adj4 brachial).ti,ab. 18 (hallux adj4 pressure).ti,ab. 19 (hallux adj4 index).ti,ab. 20 photoplethysmograph*.ti,ab. 21 or/1‐20 22 exp Peripheral Vascular Diseases/di 23 Arterial Occlusive Diseases/di 24 exp Arteriosclerosis/di 25 exp Peripheral Arterial Disease/di 26 Intermittent Claudication/di 27 (atherosclero* or arteriosclero* or PVD or PAOD or PAD).ti,ab. 28 (arter* adj4 (occlus* or steno* or obstuct* or lesio* or block* or obliter*)).ti,ab. 29 (vascular adj4 (occlus* or steno* or obstuct* or lesio* or block* or obliter* or califi*)).ti,ab. 30 (peripher* adj4 (occlus* or steno* or obstuct* or lesio* or block* or obliter*)).ti,ab. 31 (peripheral adj3 disease*).ti,ab. 32 arteriopathic.ti,ab. 33 (claudic* or hinken*).ti,ab. 34 (critical limb ischemia or CLI).ti,ab. 35 dysvascular*.ti,ab. 36 ((leg or legs) adj4 (obstruct* or occlus* or stenos* or block* or obliter*)).ti,ab. 37 ((limb or limbs) adj4 (obstruct* or occlus* or stenos* or block* or obliter*)).ti,ab. 38 (lower adj3 extremit* adj4 (obstruct* or occlus* or stenos* or block* or obliter*)).ti,ab. 39 exp Atherosclerosis/di 40 or/22‐39 41 21 and 40 42 (exp animal/ or nonhuman/) not exp human/ 43 41 not 42	Dec 2020: 9057 Aug 2021: 1212 Aug 2022:1268 February 2024: 1349
3. CINAHL via Ebsco (Date of most recent search: 27 February 2024)	S40 S21 AND S39 S39 S22 OR S23 OR S24 OR S25 OR S26 OR S27 OR S28 OR S29 OR S30 OR S31 OR S32 OR S33 OR S34 OR S35 OR S36 OR S37 OR S38 S38 (MH "Atherosclerosis/DI") S37 TX (lower N3 extremit*) N4 (obstruct* or occlus* or stenos* or block* or obliter*) S36 TX limb or limbs) N4 (obstruct* or occlus* or stenos* or block* or obliter*) S35 TX (leg or legs) N4 (obstruct* or occlus* or stenos* or block* or obliter*) S34 TX dysvascular* S33 TX critical limb ischemia or CLI S32 TX claudic* or hinken* S31 TX arteriopathic S30 TX peripheral N3 disease* S29 TX (peripher* N4 (occlus* or steno* or obstuct* or lesio* or block* or obliter*)) S28 TX (vascular N4 (occlus* or steno* or obstuct* or lesio* or block* or obliter* or califi*)) S27 TX (arter* N4 (occlus* or steno* or obstuct* or lesio* or block* or obliter*)) S26 TX atherosclero* or arteriosclero* or PVD or PAOD or PAD S25 (MH "Intermittent Claudication/DI") S24 (MH "Arteriosclerosis+/DI") S23 (MH "Arterial Occlusive Diseases+/DI") S22 (MH "Peripheral Vascular Diseases+/DI") S21 S1 OR S2 OR S3 OR S4 OR S5 OR S6 OR S7 OR S8 OR S9 OR S10 OR S11 OR S12 OR S13 OR S14 OR S15 OR S16 OR S17 OR S18 OR S19 OR S20 S20 TX photoplethysmograph* S19 TX hallux N4 index S18 TX hallux N4 pressure S17 TX hallux N4 brachial S16 TX TAI S15 TX TBPI S14 TX TBI S13 TX Toes and "Ultrasonography, Doppler, Duplex" S12 TX Toes and "blood flow velocity" S11 TX Toes and "regional blood flow" S10 TX Toes and "brachial artery" S9 (MH "Toes") S8 TX Toes and "arterial pressure" S7 TX Toes and "blood pressure" S6 TX Toes and plethysmography S5 TX Toes and systol* S4 TX (toe* N4 pressure) S3 TX (toe* N4 (index or indices)) S2 TX toe* N4 brachial S1 (MH "Ankle Brachial Index")	Dec 2020: 1812 Aug 2021: 128 Aug 2022: 104 February 2024: 89
4. Web of Science via Clarivate (Date of most recent search: 27 February 2024)	TOPIC: (peripheral arterial disease OR Peripheral Vascular Diseases OR Arterial Occlusive Diseases OR Intermittent Claudication) AND TOPIC: (Toe brachial index OR toe systolic OR Ankle Brachial Index OR photoplethysmograph*) AND TOPIC: (DIAGNOS*)	Dec 2020: 1157 Aug 2021: 170 Aug 2022: 448 February 2024: 5
5. LILACS Bireme (Date of most recent search: 27 February 2024)	peripheral arterial disease OR Peripheral Vascular Diseases OR Arterial Occlusive Diseases OR Intermittent Claudication [Words] and Toe brachial index OR toe systolic OR Ankle Brachial Index OR photoplethysmography [Words]	Dec 2020: 48 Aug 2021: 3 Aug 2022: 2 February 2024: 2
6. ClinicalTrials.gov (www.clinicaltrials.gov) (Date of most recent search: 27 February 2024)	Toe brachial index OR toe systolic OR Ankle Brachial Index OR photoplethysmography | peripheral arterial disease OR Peripheral Vascular Diseases OR Arterial Occlusive Diseases OR Intermittent Claudication	Dec 2020: 372 Aug 2021: 59 Aug 2022: 58 February 2024: 10
7. ICTRP (Date of most recent search: 27 February 2024)	Toe brachial index OR toe systolic OR Ankle Brachial Index OR photoplethysmography | peripheral arterial disease OR Peripheral Vascular Diseases OR Arterial Occlusive Diseases OR Intermittent Claudication	Dec 2020: 0 Aug 2021: 13 Aug 2022: 1 February 2024: 0
8. DARE (Database of Abstracts of Reviews of Effects) and HTA (the Health Technology Assessment Database) (Date of most recent search: 27 February 2024)	Toe brachial index OR toe systolic OR Ankle Brachial Index OR photoplethysmography | peripheral arterial disease OR Peripheral Vascular Diseases OR Arterial Occlusive Diseases OR Intermittent Claudication	Dec 2020: 519 Aug 2021: 0 Aug 2022: 0 February 2024: 0
9. British Library Zetoc conference search via Zetoc Database (Date of most recent search: 1 August 2021. Service withdrawn August 2022)	Toe brachial index OR toe systolic OR Ankle Brachial Index OR photoplethysmography	Dec 2020: 0 Aug 2021: 0 Aug 2022: 0
TOTAL before deduplication		Dec 2020: 15993 Aug 2021: 1933 Aug 2022: 2249 February 2024: 1667
TOTAL after deduplication		Dec 2020: 11977 Aug 2021: 716 Aug 2022: 1301 February 2024: 1469

### Appendix 2. QUADAS‐2

**Table:**

**Domains and Questions**	**Rating criteria**
**Domain 1: Patient selection**	
A: Description Describe methods of patient selection Describe included patients (previous testing, presentation, intended use of index test, and setting)	
B: Signalling Questions Was a consecutive or random sample of patients enrolled? Did all patients receive the same reference standard? Was a case‐control design avoided? Did the study avoid inappropriate exclusions (excluded due to health status, abnormal index test results)?	Yes, No, Unclear
C: Risk of Bias Could the selection of patients have introduced bias?	Yes, No, Unclear
D: Concerns regarding applicability (high, low, unclear) Is there concern that the included patients do not match the review question?	Yes, No, Unclear
**Domain 2: Index test**	
A: Describe the index test and how it was conducted and interpreted	
B: Signalling Questions TBI: Was the index test measured using adequately detailed, appropriately described and executed testing methodology? TSBP: Was the index test measured using adequately detailed, appropriately described and executed testing methodology? Where both TSBP and TBI measurements were taken, were the index tests interpreted blinded to each other? TBI: Were the index test results interpreted without knowledge of the results of the reference standard? TSBP: Were the index test results interpreted without knowledge of the results of the reference standard? TBI: If a threshold was used, was it prespecified? TSBP: If a threshold was used, was it prespecified?	Yes, No, Unclear
C: Risk of Bias Could the conduct or interpretation of the index test have introduced bias?	Yes, No, Unclear
D: Concerns about applicability Are there concerns that the index test, its conduct, or its interpretation differ from the review question?	Yes, No, Unclear
**Domain 3: Reference standard**	
A: Describe the reference standard and how it was conducted and interpreted	
B: Signalling Questions Is the reference standard likely to correctly classify the target condition? Were the reference standard results interpreted without knowledge of the results of the index test?	Yes, No, Unclear
C: Risk of Bias Could the reference standard, its conduct, or its interpretation have introduced bias?	Yes, No, Unclear
D: Concerns about applicability Are there concerns that the target condition as defined by the reference standard does not match the review question?	Yes, No, Unclear
**Domain 4: Flow and timing**	
A: Describe any patients who did not receive the index tests or reference standard or who were excluded from the 2×2 table Describe the interval and any interventions between the index tests and the reference standard	
B: Signalling Questions Was there an appropriate interval between index tests and reference standard? Did all patients receive a reference standard? Did all patients receive the same reference standard? Were all patients included in the analysis?	Yes, No, Unclear
C: Risk of Bias Could the patient flow have introduced bias?	Yes, No, Unclear
**Judgements for Risk of Bias for a given domain**	
If we answer all signalling questions for a domain "yes", then we will judge risk of bias as "low". If we answer all or most signalling questions for a domain "no", then we will judge risk of bias as "high". If we answer only one signalling question for a domain "no", we will further discuss the "risk of bias" judgement. If we answer all signalling questions for a domain “unclear”, then we will judge risk of bias as "unclear".	

## Data

Presented below are all the data for all of the tests entered into the review.

**Tests:** Data tables by test

Test	No. of studies	No. of participants
1 TBI	14	2045
2 TSBP	5	701

## Characteristics of studies

### Characteristics of included studies [ordered by study ID]

**Table:** AbuRahma 2020

***Study characteristics***			
Patient Sampling	Retrospective, unclear about sampling method, excluded patients who did not have symptoms		
Patient characteristics and setting	Excluded asymptomatic PAD		
Index tests	Toe‐brachial index		
Target condition and reference standard(s)	50% or greater stenosis in any arterial segment; colour duplex ultrasound		
Flow and timing	Unclear		
Comparative	Ankle brachial index		
Notes			
***Methodological quality***			
**Item**	**Authors' judgement**	**Risk of bias**	**Applicability concerns**
**DOMAIN 1: Patient selection**			
Was a consecutive or random sample of patients enrolled?	Unclear		
Was a case‐control design avoided?	Yes		
Did the study avoid inappropriate exclusions?	No		
**Could the selection of patients have introduced bias?**		High risk	
**Are there concerns that the included patients and setting do not match the review question?**			Unclear
**DOMAIN 2: Index test (All tests)**			
Were the index test results interpreted without knowledge of the results of the reference standard?	Unclear		
If a threshold was used, was it pre‐specified?	Yes		
Where both TSBP and TBI measurements were taken, were the index tests interpreted blinded to each other?			
**Could the conduct or interpretation of the index test have introduced bias?**		Unclear risk	
**Was the index test measured using an adequately detailed, appropriately described and executed testing methodology?**			
**Are there concerns that the index test, its conduct, or interpretation differ from the review question?**			Unclear
**DOMAIN 3: Reference standard**			
Is the reference standard likely to correctly classify the target condition?	Yes		
Were the reference standard results interpreted without knowledge of the results of the index tests?	Unclear		
**Could the reference standard, its conduct, or its interpretation have introduced bias?**		Unclear risk	
**Are there concerns that the target condition as defined by the reference standard does not match the question?**			Low concern
**DOMAIN 4: Flow and timing**			
Was there an appropriate interval between index test and reference standard?	Unclear		
Did all patients receive the same reference standard?	Yes		
Were all patients included in the analysis?	Yes		
**Could the patient flow have introduced bias?**		Unclear risk

**Table:** Babaei 2020

***Study characteristics***			
Patient Sampling	Cross‐sectional study with inclusion and exclusion criteria, however sampling method not clearly described		
Patient characteristics and setting	Participants with diabetes who concord with criteria for assessment of PAD according to ADA guidelines		
Index tests	Toe systolic blood pressure and toe‐brachial index		
Target condition and reference standard(s)	Peripheral arterial disease. Colour duplex ultrasound of both limbs by highly experienced vascular sonographer.		
Flow and timing	Tests performed on the same day		
Comparative	Ankle brachial index; pulse volume wave analysis		
Notes			
***Methodological quality***			
**Item**	**Authors' judgement**	**Risk of bias**	**Applicability concerns**
**DOMAIN 1: Patient selection**			
Was a consecutive or random sample of patients enrolled?	Unclear		
Was a case‐control design avoided?	Yes		
Did the study avoid inappropriate exclusions?	Yes		
**Could the selection of patients have introduced bias?**		Unclear risk	
**Are there concerns that the included patients and setting do not match the review question?**			Low concern
**DOMAIN 2: Index test (All tests)**			
Were the index test results interpreted without knowledge of the results of the reference standard?	Yes		
If a threshold was used, was it pre‐specified?	No		
Where both TSBP and TBI measurements were taken, were the index tests interpreted blinded to each other?			
**Could the conduct or interpretation of the index test have introduced bias?**		High risk	
**Was the index test measured using an adequately detailed, appropriately described and executed testing methodology?**			
**Are there concerns that the index test, its conduct, or interpretation differ from the review question?**			Low concern
**DOMAIN 3: Reference standard**			
Is the reference standard likely to correctly classify the target condition?	Yes		
Were the reference standard results interpreted without knowledge of the results of the index tests?	Yes		
**Could the reference standard, its conduct, or its interpretation have introduced bias?**		Low risk	
**Are there concerns that the target condition as defined by the reference standard does not match the question?**			Low concern
**DOMAIN 4: Flow and timing**			
Was there an appropriate interval between index test and reference standard?	Yes		
Did all patients receive the same reference standard?	Yes		
Were all patients included in the analysis?	Yes		
**Could the patient flow have introduced bias?**		Low risk

**Table:** Chen 2021

***Study characteristics***			
Patient Sampling	100 participants enrolled from 280 active participants in a larger trial. Excluded people on dialysis.		
Patient characteristics and setting	Patients with kidney disease, not undergoing dialysis. No history of vascularisation or amputation.		
Index tests	Toe‐brachial index		
Target condition and reference standard(s)	Peripheral artery disease; defined as ≥ 50% stenosis; colour duplex ultrasound		
Flow and timing	Says clinical visits, but does not state how many or time period		
Comparative	Ankle brachial index		
Notes			
***Methodological quality***			
**Item**	**Authors' judgement**	**Risk of bias**	**Applicability concerns**
**DOMAIN 1: Patient selection**			
Was a consecutive or random sample of patients enrolled?	Unclear		
Was a case‐control design avoided?	Yes		
Did the study avoid inappropriate exclusions?	Unclear		
**Could the selection of patients have introduced bias?**		Unclear risk	
**Are there concerns that the included patients and setting do not match the review question?**			Unclear
**DOMAIN 2: Index test (All tests)**			
Were the index test results interpreted without knowledge of the results of the reference standard?	Unclear		
If a threshold was used, was it pre‐specified?	Yes		
Where both TSBP and TBI measurements were taken, were the index tests interpreted blinded to each other?			
**Could the conduct or interpretation of the index test have introduced bias?**		Unclear risk	
**Was the index test measured using an adequately detailed, appropriately described and executed testing methodology?**			
**Are there concerns that the index test, its conduct, or interpretation differ from the review question?**			Low concern
**DOMAIN 3: Reference standard**			
Is the reference standard likely to correctly classify the target condition?	Yes		
Were the reference standard results interpreted without knowledge of the results of the index tests?	Unclear		
**Could the reference standard, its conduct, or its interpretation have introduced bias?**		Unclear risk	
**Are there concerns that the target condition as defined by the reference standard does not match the question?**			Unclear
**DOMAIN 4: Flow and timing**			
Was there an appropriate interval between index test and reference standard?	Unclear		
Did all patients receive the same reference standard?	Yes		
Were all patients included in the analysis?	Yes		
**Could the patient flow have introduced bias?**		Unclear risk

**Table:** Cleofort 2023

***Study characteristics***			
Patient Sampling	50 patients and 100 limbs recruited from the Vascular Medicine Department in Paris between December 2019 and May 2021		
Patient characteristics and setting	Patients over 70 with leg or foot ulcers. Excluded patients with type 1 or 2 diabetes (treated or untreated), patients with chronic renal failure defined by a GFR < 60 mL/min/1.73 m^2^ according to CKD‐EPI, patients with a history of arterial revascularisation of a lower limb or a history of lower limb amputation, patients under guardianship or curatorship, or patients who refused to participate. Sampling method unclear/not stated		
Index tests	Ankle‐brachial index, toe‐brachial index		
Target condition and reference standard(s)	Obstructive peripheral arterial disease; colour duplex ultrasound was used as the reference standard.		
Flow and timing	Tests were a combination of data collection in real time and extraction of data from clinical records.		
Comparative	Ankle‐brachial index		
Notes			
***Methodological quality***			
**Item**	**Authors' judgement**	**Risk of bias**	**Applicability concerns**
**DOMAIN 1: Patient selection**			
Was a consecutive or random sample of patients enrolled?	Unclear		
Was a case‐control design avoided?	Yes		
Did the study avoid inappropriate exclusions?	Yes		
**Could the selection of patients have introduced bias?**		Unclear risk	
**Are there concerns that the included patients and setting do not match the review question?**			Low concern
**DOMAIN 2: Index test (All tests)**			
Were the index test results interpreted without knowledge of the results of the reference standard?	Unclear		
If a threshold was used, was it pre‐specified?	Yes		
Where both TSBP and TBI measurements were taken, were the index tests interpreted blinded to each other?	Unclear		
**Could the conduct or interpretation of the index test have introduced bias?**		Unclear risk	
**Was the index test measured using an adequately detailed, appropriately described and executed testing methodology?**			
**Are there concerns that the index test, its conduct, or interpretation differ from the review question?**			Unclear
**DOMAIN 3: Reference standard**			
Is the reference standard likely to correctly classify the target condition?	Yes		
Were the reference standard results interpreted without knowledge of the results of the index tests?	Unclear		
**Could the reference standard, its conduct, or its interpretation have introduced bias?**		Unclear risk	
**Are there concerns that the target condition as defined by the reference standard does not match the question?**			Low concern
**DOMAIN 4: Flow and timing**			
Was there an appropriate interval between index test and reference standard?	Unclear		
Did all patients receive the same reference standard?	Yes		
Were all patients included in the analysis?	Yes		
**Could the patient flow have introduced bias?**		Unclear risk

**Table:** Fejfarova 2021

***Study characteristics***			
Patient Sampling	Unclear		
Patient characteristics and setting	WIfI grading was applied with only 1 to 2 included ‐ this was based on TCPO2 results. Patients needed to have TCPO2 values of 30 to 50 mmHg at rest.		
Index tests	Toe‐brachial index		
Target condition and reference standard(s)	Peripheral artery disease; haemodynamically significant disease; colour duplex ultrasound		
Flow and timing	Not stated		
Comparative	Ankle‐brachial index; transcutaneous oximetry		
Notes			
***Methodological quality***			
**Item**	**Authors' judgement**	**Risk of bias**	**Applicability concerns**
**DOMAIN 1: Patient selection**			
Was a consecutive or random sample of patients enrolled?	Unclear		
Was a case‐control design avoided?	Yes		
Did the study avoid inappropriate exclusions?	No		
**Could the selection of patients have introduced bias?**		High risk	
**Are there concerns that the included patients and setting do not match the review question?**			High
**DOMAIN 2: Index test (All tests)**			
Were the index test results interpreted without knowledge of the results of the reference standard?	Unclear		
If a threshold was used, was it pre‐specified?	No		
Where both TSBP and TBI measurements were taken, were the index tests interpreted blinded to each other?	Unclear		
**Could the conduct or interpretation of the index test have introduced bias?**		High risk	
**Was the index test measured using an adequately detailed, appropriately described and executed testing methodology?**			
**Are there concerns that the index test, its conduct, or interpretation differ from the review question?**			Unclear
**DOMAIN 3: Reference standard**			
Is the reference standard likely to correctly classify the target condition?	Unclear		
Were the reference standard results interpreted without knowledge of the results of the index tests?	Unclear		
**Could the reference standard, its conduct, or its interpretation have introduced bias?**		Unclear risk	
**Are there concerns that the target condition as defined by the reference standard does not match the question?**			Low concern
**DOMAIN 4: Flow and timing**			
Was there an appropriate interval between index test and reference standard?	Unclear		
Did all patients receive the same reference standard?	Yes		
Were all patients included in the analysis?	Yes		
**Could the patient flow have introduced bias?**		Unclear risk

**Table:** Fendrik 2023

***Study characteristics***			
Patient Sampling	Prospective, consecutive sample		
Patient characteristics and setting	> 18 attending an angiology outpatient clinic in Hungary		
Index tests	Toe‐brachial index, toe pressure		
Target condition and reference standard(s)	Peripheral artery disease; CT angiography, DS angiography, duplex ultrasound		
Flow and timing	Unclear whether tests were all completed in one session or not		
Comparative	Ankle‐brachial index; laser or automated TBI measurements		
Notes			
***Methodological quality***			
**Item**	**Authors' judgement**	**Risk of bias**	**Applicability concerns**
**DOMAIN 1: Patient selection**			
Was a consecutive or random sample of patients enrolled?	Yes		
Was a case‐control design avoided?	No		
Did the study avoid inappropriate exclusions?	Yes		
**Could the selection of patients have introduced bias?**		Unclear risk	
**Are there concerns that the included patients and setting do not match the review question?**			Unclear
**DOMAIN 2: Index test (All tests)**			
Were the index test results interpreted without knowledge of the results of the reference standard?	Unclear		
If a threshold was used, was it pre‐specified?	Yes		
Where both TSBP and TBI measurements were taken, were the index tests interpreted blinded to each other?	Unclear		
**Could the conduct or interpretation of the index test have introduced bias?**		Unclear risk	
**Was the index test measured using an adequately detailed, appropriately described and executed testing methodology?**			
**Are there concerns that the index test, its conduct, or interpretation differ from the review question?**			Low concern
**DOMAIN 3: Reference standard**			
Is the reference standard likely to correctly classify the target condition?	Yes		
Were the reference standard results interpreted without knowledge of the results of the index tests?	Unclear		
**Could the reference standard, its conduct, or its interpretation have introduced bias?**		Unclear risk	
**Are there concerns that the target condition as defined by the reference standard does not match the question?**			Low concern
**DOMAIN 4: Flow and timing**			
Was there an appropriate interval between index test and reference standard?	Unclear		
Did all patients receive the same reference standard?	No		
Were all patients included in the analysis?	Yes		
**Could the patient flow have introduced bias?**		High risk

**Table:** Machaczka 2021

***Study characteristics***			
Patient Sampling	Unclear if consecutive or random sample		
Patient characteristics and setting	Diagnosis of type 2 diabetes mellitus, age older than 18 years Exclusion criteria: age < 18 years, open wounds and defects on limbs		
Index tests	Toe‐brachial index		
Target condition and reference standard(s)	Peripheral arterial disease; stenosis > 50%, colour duplex ultrasound		
Flow and timing	Unclear		
Comparative	Ankle‐brachial index		
Notes			
***Methodological quality***			
**Item**	**Authors' judgement**	**Risk of bias**	**Applicability concerns**
**DOMAIN 1: Patient selection**			
Was a consecutive or random sample of patients enrolled?	Unclear		
Was a case‐control design avoided?	Yes		
Did the study avoid inappropriate exclusions?	Unclear		
**Could the selection of patients have introduced bias?**		Unclear risk	
**Are there concerns that the included patients and setting do not match the review question?**			Unclear
**DOMAIN 2: Index test (All tests)**			
Were the index test results interpreted without knowledge of the results of the reference standard?	Unclear		
If a threshold was used, was it pre‐specified?	Yes		
Where both TSBP and TBI measurements were taken, were the index tests interpreted blinded to each other?			
**Could the conduct or interpretation of the index test have introduced bias?**		Unclear risk	
**Was the index test measured using an adequately detailed, appropriately described and executed testing methodology?**			
**Are there concerns that the index test, its conduct, or interpretation differ from the review question?**			Low concern
**DOMAIN 3: Reference standard**			
Is the reference standard likely to correctly classify the target condition?	Yes		
Were the reference standard results interpreted without knowledge of the results of the index tests?	Unclear		
**Could the reference standard, its conduct, or its interpretation have introduced bias?**		Unclear risk	
**Are there concerns that the target condition as defined by the reference standard does not match the question?**			Low concern
**DOMAIN 4: Flow and timing**			
Was there an appropriate interval between index test and reference standard?	Unclear		
Did all patients receive the same reference standard?	Yes		
Were all patients included in the analysis?	Yes		
**Could the patient flow have introduced bias?**		Unclear risk

**Table:** Normahani 2020

***Study characteristics***			
Patient Sampling	Consecutive sample		
Patient characteristics and setting	Diabetic patients aged over 18 years attending a diabetic foot clinic. Known vascular imaging or intervention in past year excluded.		
Index tests	Toe‐brachial index		
Target condition and reference standard(s)	Peripheral arterial disease; colour duplex ultrasound		
Flow and timing	Tests were completed on the same day		
Comparative	Ankle‐brachial index, transcutaneous oximetry; PAD scan; audible and visual CW Doppler		
Notes			
***Methodological quality***			
**Item**	**Authors' judgement**	**Risk of bias**	**Applicability concerns**
**DOMAIN 1: Patient selection**			
Was a consecutive or random sample of patients enrolled?	Yes		
Was a case‐control design avoided?	Yes		
Did the study avoid inappropriate exclusions?	Yes		
**Could the selection of patients have introduced bias?**		Low risk	
**Are there concerns that the included patients and setting do not match the review question?**			Low concern
**DOMAIN 2: Index test (All tests)**			
Were the index test results interpreted without knowledge of the results of the reference standard?	Yes		
If a threshold was used, was it pre‐specified?	Yes		
Where both TSBP and TBI measurements were taken, were the index tests interpreted blinded to each other?			
**Could the conduct or interpretation of the index test have introduced bias?**		Low risk	
**Was the index test measured using an adequately detailed, appropriately described and executed testing methodology?**			
**Are there concerns that the index test, its conduct, or interpretation differ from the review question?**			Low concern
**DOMAIN 3: Reference standard**			
Is the reference standard likely to correctly classify the target condition?	Yes		
Were the reference standard results interpreted without knowledge of the results of the index tests?	Yes		
**Could the reference standard, its conduct, or its interpretation have introduced bias?**		Low risk	
**Are there concerns that the target condition as defined by the reference standard does not match the question?**			Low concern
**DOMAIN 4: Flow and timing**			
Was there an appropriate interval between index test and reference standard?	Yes		
Did all patients receive the same reference standard?	Yes		
Were all patients included in the analysis?	Yes		
**Could the patient flow have introduced bias?**		Low risk

**Table:** Okamoto 2006

***Study characteristics***			
Patient Sampling	Cross‐sectional, does not specify consecutive or random sampling		
Patient characteristics and setting	36 consenting patients with renal haemodialysis; excluded those with previous amputation		
Index tests	Toe‐brachial index		
Target condition and reference standard(s)	Peripheral arterial disease, multi‐detector row computed tomography		
Flow and timing	Unclear. All index tests were performed on the same day, but not clear when MDCT was completed.		
Comparative	Ankle‐brachial index; skin perfusion pressure; transcutaneous oximetry		
Notes			
***Methodological quality***			
**Item**	**Authors' judgement**	**Risk of bias**	**Applicability concerns**
**DOMAIN 1: Patient selection**			
Was a consecutive or random sample of patients enrolled?	Unclear		
Was a case‐control design avoided?	Yes		
Did the study avoid inappropriate exclusions?	Unclear		
**Could the selection of patients have introduced bias?**		Unclear risk	
**Are there concerns that the included patients and setting do not match the review question?**			Low concern
**DOMAIN 2: Index test (All tests)**			
Were the index test results interpreted without knowledge of the results of the reference standard?	Unclear		
If a threshold was used, was it pre‐specified?	Unclear		
Where both TSBP and TBI measurements were taken, were the index tests interpreted blinded to each other?			
**Could the conduct or interpretation of the index test have introduced bias?**		Unclear risk	
**Was the index test measured using an adequately detailed, appropriately described and executed testing methodology?**			
**Are there concerns that the index test, its conduct, or interpretation differ from the review question?**			Unclear
**DOMAIN 3: Reference standard**			
Is the reference standard likely to correctly classify the target condition?	Yes		
Were the reference standard results interpreted without knowledge of the results of the index tests?	Unclear		
**Could the reference standard, its conduct, or its interpretation have introduced bias?**		Unclear risk	
**Are there concerns that the target condition as defined by the reference standard does not match the question?**			Low concern
**DOMAIN 4: Flow and timing**			
Was there an appropriate interval between index test and reference standard?	Unclear		
Did all patients receive the same reference standard?	Yes		
Were all patients included in the analysis?	Yes		
**Could the patient flow have introduced bias?**		Unclear risk

**Table:** Park 2012

***Study characteristics***			
Patient Sampling	Convenience sample, unclear how patients were recruited ‐ if consecutive or random. Selected patients with gangrene or claudication.		
Patient characteristics and setting	Population represents those heavily diseased. Patients with either diabetic gangrene or symptoms of intermittent claudication were included in the study.		
Index tests	In patients with suspected lower extremity arterial insufficiency, TBI was measured.		
Target condition and reference standard(s)	Peripheral arterial disease; angiography		
Flow and timing	All tests were performed on the same day. This is not entirely clear: "In patients diagnosed with arterial insufficiency, vessel dilation using a balloon catheter was performed. Patients with high TBI values were deemed to be candidates for this procedure." It appears there may have been some exclusions, but this is not reported.		
Comparative	Ankle‐brachial index		
Notes			
***Methodological quality***			
**Item**	**Authors' judgement**	**Risk of bias**	**Applicability concerns**
**DOMAIN 1: Patient selection**			
Was a consecutive or random sample of patients enrolled?	Unclear		
Was a case‐control design avoided?	Yes		
Did the study avoid inappropriate exclusions?	Unclear		
**Could the selection of patients have introduced bias?**		Unclear risk	
**Are there concerns that the included patients and setting do not match the review question?**			High
**DOMAIN 2: Index test (All tests)**			
Were the index test results interpreted without knowledge of the results of the reference standard?	Unclear		
If a threshold was used, was it pre‐specified?	Yes		
Where both TSBP and TBI measurements were taken, were the index tests interpreted blinded to each other?			
**Could the conduct or interpretation of the index test have introduced bias?**		Unclear risk	
**Was the index test measured using an adequately detailed, appropriately described and executed testing methodology?**			
**Are there concerns that the index test, its conduct, or interpretation differ from the review question?**			Unclear
**DOMAIN 3: Reference standard**			
Is the reference standard likely to correctly classify the target condition?	Yes		
Were the reference standard results interpreted without knowledge of the results of the index tests?	Unclear		
**Could the reference standard, its conduct, or its interpretation have introduced bias?**		Unclear risk	
**Are there concerns that the target condition as defined by the reference standard does not match the question?**			Unclear
**DOMAIN 4: Flow and timing**			
Was there an appropriate interval between index test and reference standard?	Yes		
Did all patients receive the same reference standard?	Unclear		
Were all patients included in the analysis?	Yes		
**Could the patient flow have introduced bias?**		Unclear risk

**Table:** Singhania 2024

***Study characteristics***			
Patient Sampling	Sampling method unclear		
Patient characteristics and setting	Included patients with type 2 diabetes aged over 50 years. Excluded patients who were current or ex‐smokers, patients with contraindications to ankle or toe pressure measurement, such as those with active great toe infection or ulceration, absent great toe, vasospastic disorders, cardiac arrhythmias, and history of deep vein thrombosis or lymphoedema from the study. Patients with CKD as determined by eGFR < 60 mL/min/1.73 m^2^ were also excluded.		
Index tests	Ankle‐brachial index and toe‐brachial index		
Target condition and reference standard(s)	Peripheral arterial disease: presence of stenosis > 50%; CT angiography		
Flow and timing	Patients attended testing session. Unclear on timing and flow in relation to CT angiography.		
Comparative	Ankle‐brachial index		
Notes			
***Methodological quality***			
**Item**	**Authors' judgement**	**Risk of bias**	**Applicability concerns**
**DOMAIN 1: Patient selection**			
Was a consecutive or random sample of patients enrolled?	Unclear		
Was a case‐control design avoided?	Yes		
Did the study avoid inappropriate exclusions?	Yes		
**Could the selection of patients have introduced bias?**		Unclear risk	
**Are there concerns that the included patients and setting do not match the review question?**			Low concern
**DOMAIN 2: Index test (All tests)**			
Were the index test results interpreted without knowledge of the results of the reference standard?	Unclear		
If a threshold was used, was it pre‐specified?	Yes		
Where both TSBP and TBI measurements were taken, were the index tests interpreted blinded to each other?	Unclear		
**Could the conduct or interpretation of the index test have introduced bias?**		Unclear risk	
**Was the index test measured using an adequately detailed, appropriately described and executed testing methodology?**			
**Are there concerns that the index test, its conduct, or interpretation differ from the review question?**			Low concern
**DOMAIN 3: Reference standard**			
Is the reference standard likely to correctly classify the target condition?	Yes		
Were the reference standard results interpreted without knowledge of the results of the index tests?	Unclear		
**Could the reference standard, its conduct, or its interpretation have introduced bias?**		Unclear risk	
**Are there concerns that the target condition as defined by the reference standard does not match the question?**			Low concern
**DOMAIN 4: Flow and timing**			
Was there an appropriate interval between index test and reference standard?	Unclear		
Did all patients receive the same reference standard?	Yes		
Were all patients included in the analysis?	Yes		
**Could the patient flow have introduced bias?**		Unclear risk

**Table:** Sonter 2017

***Study characteristics***			
Patient Sampling	Convenience sample		
Patient characteristics and setting	Podiatry clinic and community; people aged over 50 years with a history of diabetes or smoking, or any person aged over 65 years		
Index tests	Automated toe systolic blood pressure and toe‐brachial index		
Target condition and reference standard(s)	Peripheral artery disease; defined as one or more arteries with > 50% stenosis; colour duplex ultrasound		
Flow and timing	Same day		
Comparative	Nil		
Notes			
***Methodological quality***			
**Item**	**Authors' judgement**	**Risk of bias**	**Applicability concerns**
**DOMAIN 1: Patient selection**			
Was a consecutive or random sample of patients enrolled?	Unclear		
Was a case‐control design avoided?	Yes		
Did the study avoid inappropriate exclusions?	Yes		
**Could the selection of patients have introduced bias?**		Unclear risk	
**Are there concerns that the included patients and setting do not match the review question?**			Low concern
**DOMAIN 2: Index test (All tests)**			
Were the index test results interpreted without knowledge of the results of the reference standard?	Unclear		
If a threshold was used, was it pre‐specified?	Yes		
Where both TSBP and TBI measurements were taken, were the index tests interpreted blinded to each other?	Unclear		
**Could the conduct or interpretation of the index test have introduced bias?**		Unclear risk	
**Was the index test measured using an adequately detailed, appropriately described and executed testing methodology?**			
**Are there concerns that the index test, its conduct, or interpretation differ from the review question?**			Low concern
**DOMAIN 3: Reference standard**			
Is the reference standard likely to correctly classify the target condition?	Yes		
Were the reference standard results interpreted without knowledge of the results of the index tests?	Unclear		
**Could the reference standard, its conduct, or its interpretation have introduced bias?**		Unclear risk	
**Are there concerns that the target condition as defined by the reference standard does not match the question?**			Low concern
**DOMAIN 4: Flow and timing**			
Was there an appropriate interval between index test and reference standard?	Yes		
Did all patients receive the same reference standard?	Yes		
Were all patients included in the analysis?	Yes		
**Could the patient flow have introduced bias?**		Low risk

**Table:** Tehan 2016

***Study characteristics***			
Patient Sampling	Convenience sample		
Patient characteristics and setting	Patients who met AHA criteria for vascular assessment or with symptoms of suspected PAD		
Index tests	Toe‐brachial index		
Target condition and reference standard(s)	Peripheral artery disease; > 50% stenosis; colour duplex ultrasound		
Flow and timing	Test performed on the same day		
Comparative	Ankle‐brachial index; continuous wave Doppler		
Notes			
***Methodological quality***			
**Item**	**Authors' judgement**	**Risk of bias**	**Applicability concerns**
**DOMAIN 1: Patient selection**			
Was a consecutive or random sample of patients enrolled?	Unclear		
Was a case‐control design avoided?	Yes		
Did the study avoid inappropriate exclusions?	Yes		
**Could the selection of patients have introduced bias?**		Unclear risk	
**Are there concerns that the included patients and setting do not match the review question?**			Low concern
**DOMAIN 2: Index test (All tests)**			
Were the index test results interpreted without knowledge of the results of the reference standard?	No		
If a threshold was used, was it pre‐specified?	Yes		
Where both TSBP and TBI measurements were taken, were the index tests interpreted blinded to each other?			
**Could the conduct or interpretation of the index test have introduced bias?**		Unclear risk	
**Was the index test measured using an adequately detailed, appropriately described and executed testing methodology?**			
**Are there concerns that the index test, its conduct, or interpretation differ from the review question?**			Low concern
**DOMAIN 3: Reference standard**			
Is the reference standard likely to correctly classify the target condition?	Yes		
Were the reference standard results interpreted without knowledge of the results of the index tests?	No		
**Could the reference standard, its conduct, or its interpretation have introduced bias?**		Unclear risk	
**Are there concerns that the target condition as defined by the reference standard does not match the question?**			Low concern
**DOMAIN 4: Flow and timing**			
Was there an appropriate interval between index test and reference standard?	Yes		
Did all patients receive the same reference standard?	Yes		
Were all patients included in the analysis?	Yes		
**Could the patient flow have introduced bias?**		Low risk

**Table:** Tehan 2017

***Study characteristics***			
Patient Sampling	Consecutive patient records. Title indicates case‐control, however no controls, simply diabetes and no diabetes (target condition in both groups).		
Patient characteristics and setting	Patients attending lab for suspected PAD (symptoms or review, known PAD)		
Index tests	Toe systolic blood pressure		
Target condition and reference standard(s)	Peripheral artery disease; > 50% stenosis in any vessel; colour duplex ultrasound		
Flow and timing	Index tests performed first, followed by reference standard		
Comparative	Diabetes and no diabetes; no index test comparator		
Notes			
***Methodological quality***			
**Item**	**Authors' judgement**	**Risk of bias**	**Applicability concerns**
**DOMAIN 1: Patient selection**			
Was a consecutive or random sample of patients enrolled?	Yes		
Was a case‐control design avoided?	Yes		
Did the study avoid inappropriate exclusions?	Yes		
**Could the selection of patients have introduced bias?**		Low risk	
**Are there concerns that the included patients and setting do not match the review question?**			Low concern
**DOMAIN 2: Index test (All tests)**			
Were the index test results interpreted without knowledge of the results of the reference standard?	Unclear		
If a threshold was used, was it pre‐specified?	No		
Where both TSBP and TBI measurements were taken, were the index tests interpreted blinded to each other?			
**Could the conduct or interpretation of the index test have introduced bias?**		High risk	
**Was the index test measured using an adequately detailed, appropriately described and executed testing methodology?**			
**Are there concerns that the index test, its conduct, or interpretation differ from the review question?**			Low concern
**DOMAIN 3: Reference standard**			
Is the reference standard likely to correctly classify the target condition?	Yes		
Were the reference standard results interpreted without knowledge of the results of the index tests?	Unclear		
**Could the reference standard, its conduct, or its interpretation have introduced bias?**		Unclear risk	
**Are there concerns that the target condition as defined by the reference standard does not match the question?**			Low concern
**DOMAIN 4: Flow and timing**			
Was there an appropriate interval between index test and reference standard?	Unclear		
Did all patients receive the same reference standard?	Yes		
Were all patients included in the analysis?	Yes		
**Could the patient flow have introduced bias?**		Unclear risk

**Table:** Tehan 2021

***Study characteristics***			
Patient Sampling	Convenience		
Patient characteristics and setting	Adults exhibiting signs or symptoms consistent with PAD were recruited. Vascular laboratory setting.		
Index tests	Toe‐brachial index and toe systolic pressure		
Target condition and reference standard(s)	Peripheral arterial disease, > 50% stenosis; colour duplex ultrasound		
Flow and timing	Same day		
Comparative	Ankle‐brachial index		
Notes			
***Methodological quality***			
**Item**	**Authors' judgement**	**Risk of bias**	**Applicability concerns**
**DOMAIN 1: Patient selection**			
Was a consecutive or random sample of patients enrolled?	Unclear		
Was a case‐control design avoided?	Yes		
Did the study avoid inappropriate exclusions?	Yes		
**Could the selection of patients have introduced bias?**		Unclear risk	
**Are there concerns that the included patients and setting do not match the review question?**			Low concern
**DOMAIN 2: Index test (All tests)**			
Were the index test results interpreted without knowledge of the results of the reference standard?	Unclear		
If a threshold was used, was it pre‐specified?	Yes		
Where both TSBP and TBI measurements were taken, were the index tests interpreted blinded to each other?	Unclear		
**Could the conduct or interpretation of the index test have introduced bias?**		Unclear risk	
**Was the index test measured using an adequately detailed, appropriately described and executed testing methodology?**			
**Are there concerns that the index test, its conduct, or interpretation differ from the review question?**			Low concern
**DOMAIN 3: Reference standard**			
Is the reference standard likely to correctly classify the target condition?	Yes		
Were the reference standard results interpreted without knowledge of the results of the index tests?	Unclear		
**Could the reference standard, its conduct, or its interpretation have introduced bias?**		Unclear risk	
**Are there concerns that the target condition as defined by the reference standard does not match the question?**			Low concern
**DOMAIN 4: Flow and timing**			
Was there an appropriate interval between index test and reference standard?	Yes		
Did all patients receive the same reference standard?	Yes		
Were all patients included in the analysis?	Yes		
**Could the patient flow have introduced bias?**		Low risk

**Table:** Tsuyuki 2013

***Study characteristics***			
Patient Sampling	Convenience sample most likely		
Patient characteristics and setting	26 ambulatory haemodialysis patients (19 men and 9 women). 10 patients with diabetes mellitus. Excluded patients with a history of amputation.		
Index tests	Toe‐brachial index		
Target condition and reference standard(s)	Computed tomographic angiography (CTA) was performed in 16 patients (32 limbs) who gave informed consent on a non‐HD day. Peripheral arterial disease target condition.		
Flow and timing	This is not clearly stated. Although it states that the CTA scan was performed on a non‐HD day, it does not then state when the index tests were performed. 32 limbs had CTA.		
Comparative	Ankle‐brachial index; resting and post‐exercise values; ankle pressures		
Notes			
***Methodological quality***			
**Item**	**Authors' judgement**	**Risk of bias**	**Applicability concerns**
**DOMAIN 1: Patient selection**			
Was a consecutive or random sample of patients enrolled?	Unclear		
Was a case‐control design avoided?	Yes		
Did the study avoid inappropriate exclusions?	Unclear		
**Could the selection of patients have introduced bias?**		Unclear risk	
**Are there concerns that the included patients and setting do not match the review question?**			Low concern
**DOMAIN 2: Index test (All tests)**			
Were the index test results interpreted without knowledge of the results of the reference standard?	Unclear		
If a threshold was used, was it pre‐specified?	Yes		
Where both TSBP and TBI measurements were taken, were the index tests interpreted blinded to each other?			
**Could the conduct or interpretation of the index test have introduced bias?**		Unclear risk	
**Was the index test measured using an adequately detailed, appropriately described and executed testing methodology?**			
**Are there concerns that the index test, its conduct, or interpretation differ from the review question?**			Low concern
**DOMAIN 3: Reference standard**			
Is the reference standard likely to correctly classify the target condition?	Yes		
Were the reference standard results interpreted without knowledge of the results of the index tests?	Unclear		
**Could the reference standard, its conduct, or its interpretation have introduced bias?**		Unclear risk	
**Are there concerns that the target condition as defined by the reference standard does not match the question?**			Low concern
**DOMAIN 4: Flow and timing**			
Was there an appropriate interval between index test and reference standard?	Unclear		
Did all patients receive the same reference standard?	Yes		
Were all patients included in the analysis?	Yes		
**Could the patient flow have introduced bias?**		Unclear risk

**Table:** Vriens 2018

***Study characteristics***			
Patient Sampling	Convenience sample (?), not stated; during routine clinical visits, patients with new ulceration and no history of revascularisation		
Patient characteristics and setting	Peripheral arterial disease. All individuals with diabetes, regardless of type, presenting with primary lower limb ulceration were potentially eligible.		
Index tests	Toe pressures and toe‐brachial index		
Target condition and reference standard(s)	Peripheral artery disease; PSV ratio > 2 equivalent to > 50% stenosis; duplex ultrasound scan		
Flow and timing	Timing not specified but likely multiple visits, as it states that tests were completed during routine clinical visits.		
Comparative	Pedal pulses, hair loss, atrophy, dependent rubor, cool skin, blue/purple skin; capillary refill; venous filling; ankle pressure; toe pressure; ankle‐brachial index; pulse test at ankle; TcP02; waveform analysis		
Notes			
***Methodological quality***			
**Item**	**Authors' judgement**	**Risk of bias**	**Applicability concerns**
**DOMAIN 1: Patient selection**			
Was a consecutive or random sample of patients enrolled?	Unclear		
Was a case‐control design avoided?	Yes		
Did the study avoid inappropriate exclusions?	Unclear		
**Could the selection of patients have introduced bias?**		Unclear risk	
**Are there concerns that the included patients and setting do not match the review question?**			Low concern
**DOMAIN 2: Index test (All tests)**			
Were the index test results interpreted without knowledge of the results of the reference standard?	Yes		
If a threshold was used, was it pre‐specified?	Yes		
Where both TSBP and TBI measurements were taken, were the index tests interpreted blinded to each other?	Unclear		
**Could the conduct or interpretation of the index test have introduced bias?**		Low risk	
**Was the index test measured using an adequately detailed, appropriately described and executed testing methodology?**			
**Are there concerns that the index test, its conduct, or interpretation differ from the review question?**			Low concern
**DOMAIN 3: Reference standard**			
Is the reference standard likely to correctly classify the target condition?	Yes		
Were the reference standard results interpreted without knowledge of the results of the index tests?	Yes		
**Could the reference standard, its conduct, or its interpretation have introduced bias?**		Low risk	
**Are there concerns that the target condition as defined by the reference standard does not match the question?**			Low concern
**DOMAIN 4: Flow and timing**			
Was there an appropriate interval between index test and reference standard?	Unclear		
Did all patients receive the same reference standard?	Yes		
Were all patients included in the analysis?	Yes		
**Could the patient flow have introduced bias?**		Unclear risk

**Table:** Williams 2005

***Study characteristics***			
Patient Sampling	Volunteer sample. Did not include patients with active foot disease or CLI (end stage PAD). Excluded smokers and renal disease.		
Patient characteristics and setting	68 volunteer participants with type 1 or 2 diabetes. No individuals had active foot disease, rest pain or signs suggestive of lower‐limb critical ischaemia.		
Index tests	Toe‐brachial index		
Target condition and reference standard(s)	Peripheral arterial disease, colour duplex imaging		
Flow and timing	All tests performed on the same day		
Comparative	Foot pulses; ankle‐brachial index; CW Doppler; diabetes and control groups		
Notes			
***Methodological quality***			
**Item**	**Authors' judgement**	**Risk of bias**	**Applicability concerns**
**DOMAIN 1: Patient selection**			
Was a consecutive or random sample of patients enrolled?	Unclear		
Was a case‐control design avoided?	No		
Did the study avoid inappropriate exclusions?	No		
**Could the selection of patients have introduced bias?**		High risk	
**Are there concerns that the included patients and setting do not match the review question?**			Unclear
**DOMAIN 2: Index test (All tests)**			
Were the index test results interpreted without knowledge of the results of the reference standard?	Unclear		
If a threshold was used, was it pre‐specified?	Yes		
Where both TSBP and TBI measurements were taken, were the index tests interpreted blinded to each other?			
**Could the conduct or interpretation of the index test have introduced bias?**		Unclear risk	
**Was the index test measured using an adequately detailed, appropriately described and executed testing methodology?**			
**Are there concerns that the index test, its conduct, or interpretation differ from the review question?**			Low concern
**DOMAIN 3: Reference standard**			
Is the reference standard likely to correctly classify the target condition?	Yes		
Were the reference standard results interpreted without knowledge of the results of the index tests?	Unclear		
**Could the reference standard, its conduct, or its interpretation have introduced bias?**		Unclear risk	
**Are there concerns that the target condition as defined by the reference standard does not match the question?**			Low concern
**DOMAIN 4: Flow and timing**			
Was there an appropriate interval between index test and reference standard?	Yes		
Did all patients receive the same reference standard?	Yes		
Were all patients included in the analysis?	Yes		
**Could the patient flow have introduced bias?**		Low risk	

ADA: American Diabetes Association AHA: American Heart Association CKD: chronic kidney disease CKD‐EPI: Chronic Kidney Disease Epidemiology Collaboration CLI: critical limb ischaemia CT: computed tomography CTA: computed tomographic angiography CW: continuous wave DS: digital subtraction (e)GFR: (estimated) glomerular filtration rate HD: haemodialysis MDCT: multi‐detector computed tomography  non‐HD: non‐haemodialysis PAD: peripheral arterial disease TBI: toe‐brachial index TCPO2: transcutaneous oxygen pressure WIfI: Wound, Ischaemia, and foot Infection

### Characteristics of excluded studies [ordered by study ID]

**Table:**

Study	Reason for exclusion
Aboyans 2008	Wrong reference standard
Ali 2020	Wrong comparator
Alnaeb 2007	Wrong comparator
Alvaro‐Afonso 2018	Wrong study design (not diagnostic test accuracy)
Arfvidsson 1992	Wrong study design (not diagnostic test accuracy)
Armstrong 2011	Wrong study design (not diagnostic test accuracy)
Armstrong 2013	Wrong study design (not diagnostic test accuracy)
Armstrong 2013a	Wrong study design (not diagnostic test accuracy)
Armstrong 2021	Wrong study design (not diagnostic test accuracy)
Arora 2020	Wrong study design (not diagnostic test accuracy)
Aursulesei 2013	Unable to contact authors, abstract only
Baloch 2022	Inappropriate time between index and reference standard (> 1 month)
Bird 1999	Wrong study design (not diagnostic test accuracy)
Boccalon 1992	Wrong study design (not diagnostic test accuracy)
Bonham 2006	Wrong study design (not diagnostic test accuracy)
Camilleri 2021	Wrong study design (not diagnostic test accuracy)
Carter 1973	Wrong study design (not diagnostic test accuracy)
Carter 1996	Wrong reference standard
Carter 2001	Wrong study design (not diagnostic test accuracy)
Chandarana 2018	Wrong study design (not diagnostic test accuracy)
Chantelau 1996	Wrong study design (not diagnostic test accuracy)
Choi 2023	Wrong reference standard
Chou 2020	Wrong study design (not diagnostic test accuracy)
Chuter 2014	Duplicate data
Chuter 2018	Wrong study design (not diagnostic test accuracy)
Cohen 2014	Wrong study design (not diagnostic test accuracy)
Criqui 1996	Wrong study design (not diagnostic test accuracy)
de Athayde 2021	Wrong study design (not diagnostic test accuracy)
De Graaff 2003	Reference test not in all participants
Di Nardo 1984	Wrong study design (not diagnostic test accuracy)
Ding 2015	Wrong study design (not diagnostic test accuracy)
Eickhoff 1980	Reference test not in all participants
Elewa 2014	Wrong reference standard
Fejfarova 2018	Unable to contact authors ‐ suspected duplicate data
Fendrik 2023a	Unable to contact authors
Friethriksson 2005	Wrong study design (not diagnostic test accuracy)
Glargaard 2019	Wrong reference standard
Glargaard 2020	Wrong reference standard
Goyal 2013	Wrong comparator
Gundersen 1971	Wrong study design (not diagnostic test accuracy)
Gutierrez 1981	Wrong reference standard
He 2012	Unable to contact authors for data
Hirai 1982	Wrong study design (not diagnostic test accuracy)
Hoffmann 2019	Wrong outcomes
Horstick 2017	Wrong index test
Horstick 2020	Wrong index test
Hoyer 2013a	Wrong reference standard
Hoyer 2013b	Wrong outcomes
Igari 2014	Wrong reference standard
Igari 2014a	Wrong study design (not diagnostic test accuracy)
Jacobs 1990	Wrong study design (not diagnostic test accuracy)
Jogestrand 1983	Wrong reference standard
Karunakaran 2016	Wrong study design (not diagnostic test accuracy)
Kawamoto 2012	Wrong study design (not diagnostic test accuracy)
Koivisto 1988	Wrong study design (not diagnostic test accuracy)
Kovacs 2018	Wrong reference standard
Kusumanto 2006	Wrong study design (not diagnostic test accuracy)
Laivuori 2021	Wrong study design (not diagnostic test accuracy)
Lanting 2021	Wrong study design (not diagnostic test accuracy)
Larsen 1990	Wrong study design (not diagnostic test accuracy)
Lassen 1975	Wrong study design (not diagnostic accuracy)
Lepantalo 1988	Wrong reference standard
Leskinen 2002	Reference test not in all participants
Lurie 2021a	Wrong study design (not diagnostic accuracy)
Lurie 2021b	Wrong study design (not diagnostic test accuracy)
Manu 2021	Wrong study design (not diagnostic test accuracy)
Manu 2022	Wrong reference standard
Martin Borge 2008	Wrong reference standard
Matsumura 2013	Wrong study design (not diagnostic test accuracy)
Mizzi 2019	Wrong study design (not diagnostic test accuracy)
Mizzi 2020	Wrong study design (not diagnostic test accuracy)
Murray 2012	Wrong reference standard
Mwipatayi 2005	Wrong study design (not diagnostic test accuracy)
Nattero‐Chavez 2019	Wrong comparator
Ng 2019	Wrong study design (not diagnostic test accuracy)
Nicolson 2010	Wrong study design (not diagnostic test accuracy)
Nielsen 1989	Wrong study design (not diagnostic test accuracy)
Nikulainen 2020	Wrong study design (not diagnostic test accuracy)
Normahani 2021	Wrong study design (not diagnostic test accuracy)
Nuttawut 2021	Wrong study design (not diagnostic test accuracy)
Ohtake 2011	Wrong reference standard
Ostrowski 1974	Wrong reference standard
Papanas 2004	Wrong reference standard
Parving 1996	Wrong study design (not diagnostic test accuracy)
Peltokangas 2018	Wrong study design (not diagnostic test accuracy)
Peltokangas 2019	Wrong study design (not diagnostic test accuracy)
Pesonen 2018	Wrong study design (not diagnostic test accuracy)
Porciuncula 2007	Wrong study design (not diagnostic test accuracy)
Randhawa 2017	Wrong study design (not diagnostic test accuracy)
Razavi 2021	Wrong reference standard
Rheeder 2004	Wrong reference standard
Ruzsa 2015	Wrong reference standard
Sambraus 1996	Wrong reference standard
Sarlak 2013	Wrong reference standard
Scissons 2010	Wrong study design (not diagnostic test accuracy)
Settembre 2017	Wrong study design (not diagnostic test accuracy)
Shafe 2020	Wrong study design (not diagnostic test accuracy)
Soro 1985	Wrong patient population
Srivishnuprasath 2020	Wrong reference standard
Suominen 2008	Wrong study design (not diagnostic test accuracy)
Svensson 2008	Wrong reference standard
Tehan 2015	Duplicate data
Tehan 2016b	Duplicate data
Tisserand 2017	Wrong study design (not diagnostic test accuracy)
Tonnesen 1980	Wrong outcomes
Trihan 2021	Wrong outcomes
Varghese 2021	Wrong study design
Vincent 1983	Wrong reference standard
Wang 2019	Wrong study design (not diagnostic test accuracy)
Wen 2011	Wrong study design
Wickstrom 2017	Wrong study design
Wickstrom 2019	Wrong study design (not diagnostic test accuracy)
Widmer 2012	Wrong study design (not diagnostic test accuracy)
Williams 2006	Duplicate data
Wlodarczyk 2019	Wrong study design (not diagnostic test accuracy)
Wukich 2015	Wrong reference standard
Yamada 2008	Wrong reference standard
Young 2016	Wrong study design (not diagnostic test accuracy)
Zaki 2016	Wrong study design (not diagnostic test accuracy)
Zierfuss 2017	Abstract only from conference, unable to obtain adequate information
Zwicky 2002	Wrong study design (not diagnostic test accuracy)

## Differences between protocol and review

Some additional members of the research team were recruited to assist with this review due to the large volume of work that was required and the competing demands of clinical practice in a pandemic (BP and SL). Morgan Hawes (MH), a research assistant, completed screening of titles and abstracts independently.

Due to the heterogeneity of the included studies, the planned meta‐analysis and other sensitivity analyses could not be performed.
